# A Vision toward Ultimate Optical Out‐Coupling for Organic Light‐Emitting Diode Displays: 3D Pixel Configuration

**DOI:** 10.1002/advs.201800467

**Published:** 2018-08-29

**Authors:** Yi‐Jiun Chen, Wei‐Kai Lee, Yi‐Ting Chen, Chun‐Yu Lin, Sheng‐Wen Wen, Min Jiao, Guo‐Dong Su, Hoang Yan Lin, Robert J. Visser, Byungsung Leo Kwak, Chung‐Chia Chen, Wan‐Yu Lin, Steve Wang, Chorng‐Ping Chang, Chung‐Chih Wu

**Affiliations:** ^1^ Graduate Institute of Photonics and Optoelectronics Graduate Institute of Electronics Engineering Department of Electrical Engineering National Taiwan University Taipei 106 Taiwan; ^2^ Applied Materials, Inc. Santa Clara, CA 95052 USA

**Keywords:** displays, light extraction, multiscale optics, organic light‐emitting diodes (OLEDs)

## Abstract

Despite stringent power consumption requirements in many applications, over years organic light‐emitting diode (OLED) displays still suffer unsatisfactory energy efficiency due to poor light extraction. Approaches have been reported for OLED light out‐coupling, but they in general are not applicable for OLED displays due to difficulties in display image quality and fabrication complexity and compatibility. Thus to date, an effective and feasible light extraction technique that can boost efficiencies and yet keep image quality is still lacking and remains a great challenge. Here, a highly effective and scalable extraction‐enhancing OLED display pixel structure is proposed based on embedding the OLED inside a three‐dimensional reflective concave structure covered with a patterned high‐index filler. It can couple as much internal emission as possible into the filler region and then redirect otherwise confined light for out‐coupling. Comprehensive multi‐scale optical simulation validates that ultimately high light extraction efficiency approaching ≈80% and excellent viewing characteristics are simultaneously achievable with optimized structures using highly transparent top electrodes. This scheme is scalable and wavelength insensitive, and generally applicable to all red, green, and blue pixels in high‐resolution full‐color displays. Results of this work are believed to shed light on the development of future generations of advanced OLED displays.

Active‐matrix organic light‐emitting diode displays (AMOLEDs) have become one of the major display technologies, due to their various attractive merits for display applications. These include excellent viewing characteristics resulting from their self‐emissive nature, vivid and saturated colors, high contrast, fast response, wide operation temperatures, compatibility with flexible and wearable applications, etc.^1^, ^2^, ^3^, ^4^, ^5^ For many AMOLED applications (e.g., mobile and wearable applications), the power consumption is a critical issue and thus achieving the highest possible external quantum efficiency (EQE) of pixel organic light‐emitting diodes (OLEDs) is essential. The EQE of an OLED is governed by both the internal quantum efficiency (IQE) of charge‐to‐photon conversion in the device and the extraction efficiency of internally generated light for external viewing. Substantial progresses have been made in developing emitting materials with high IQE, such as phosphorescent and other triplet‐harvesting emitters that can give nearly ideal 100% IQE.^6^, ^7^ However, current AMOLEDs still suffer poor light extraction and far from ideal EQEs. **Figure** **1**a shows the schematic configuration for an OLED pixel in a typical AMOLED, which usually adopts a top‐emitting configuration for a larger emitting aperture.^3^, ^5^ On top of the substrate and the thin‐film transistor circuitry, the OLED pixel in general has a bottom reflective electrode (e.g., Ag), a thick dielectric bank layer (usually a few µm thick) with tapered banks around the emitting aperture for planarization and isolation, OLED active organic layer(s), and top (semi‐)transparent electrode (e.g., thin Ag or indium tin oxide (ITO)).^8^, ^9^, ^10^ In some cases, the top (semi‐)transparent electrode may be further overcoated with a thin transparent capping/passivation layer.^8^, ^9^, ^10^ Due to higher than unity refractive indices (*n*) and optical losses in OLED organic layers (typically *n* > 1.7), transparent electrodes (typically *n* > 1.8), metallic electrodes, and capping/passivation layers, only less than 20% of generated photons can be out‐coupled for viewing in current AMOLEDs. More than 80% of generated photons remain trapped or lost within the device—either guided in the organic/dielectric/transparent electrode/capping layers (waveguided modes, WG) or bound as surface plasmon polaritons (SPPs) at the metallic contact.^11^, ^12^, ^13^ Thus, despite the high IQEs of the latest triplet‐harvesting OLEDs, optimization of light extraction provides ample room for performance improvements of AMOLEDs.

**Figure 1:**
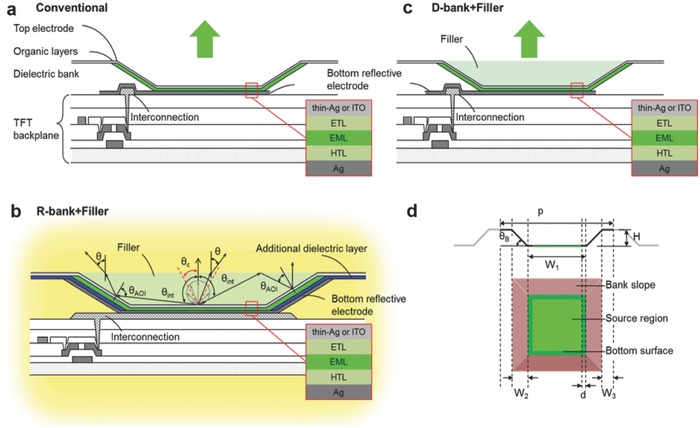
Pixel and device configurations. a) The conventional AMOLED pixel structure with a flat bottom reflective electrode (Ag) and the surrounding dielectric bank. b) The proposed 3D AMOLED pixel configuration with the selective high‐index filler and the bottom reflective electrode (Ag) being extended to the bank slope to form the concave reflector. Different light ray paths illustrate how light rays entering the filler with an initial internal angle θ_int_ exceeding θ_c_ (the total‐internal‐reflection—TIR critical angle of the filler–air interface) can be redirected for out‐coupling via one or multiple reflection by various reflective surfaces. This structure is called the R‐bank + filler structure, in which an additional dielectric layer coated over the bank slope portion of the bottom reflective electrode may be needed for insulation and for defining the emission aperture at the bottom surface. c) The AMOLED pixel structure similar to the conventional pixel structure except for the addition of the high‐index filler, called the D‐bank + filler structure herein. d) The side view and top view of the pixel structure and feature/dimension definitions for optical ray‐tracing simulation, including the bank height H, the bank angle θ_B_, the bottom width *W*_1_ of the concave structure, the spacing *d* between the actual emission area and the bank edge, and the subpixel pitch *p* = *W*_1_ + 2*W*_2_ + 2*W*_3_. Insets in panels (a)–(c) show more details of the active layer stack of the OLED with either thin Ag or ITO as the top electrode. Depending on whether thin Ag or ITO is adopted for the top electrode and whether the bank slope surface is coated with reflective Ag, four different structures: thin Ag + R‐bank, thin Ag + D‐bank, ITO + R‐bank, and ITO + D‐bank are studied for light extraction and viewing characteristics.

Approaches have been reported for enhancing light out‐coupling of OLEDs, such as microlens arrays,^14^, ^15^, ^16^ surface textures,^17^ scattering media,^18^, ^19^, ^20^ embedded low‐index grids,^21^, ^22^, ^23^ embedded photonic nanostructures (e.g., grating/corrugation/photonic crystals),^24^, ^25^, ^26^, ^27^, ^28^, ^29^ and high‐index substrates.^12^, ^15^, ^30^, ^31^ Although these different methods/structures may be useful for OLED lighting and/or bottom‐emitting OLED structures, they in general are not readily applicable for light extraction of (top‐emitting) AMOLED displays, mainly due to several difficulties associated with display image quality, fabrication complexity, and integration compatibility: 1) the out‐coupling structures/effects often lead to leakage/diffusion of pixel emission to neighboring pixels, resulting in pixel blurring that would degrade the display resolution and image quality;^14^, ^16^, ^19^ 2) the out‐coupling structures/effects often cause scattering, diffusive and diffractive optical reflection of incident ambient light and thus degrade display contrast and image quality;^14^, ^16^, ^17^, ^18^, ^19^, ^20^, ^24^, ^25^, ^26^, ^27^, ^28^ 3) the optical out‐coupling structures may require advanced and expensive fabrication (e.g., high‐resolution nanofabrication) not so compatible with OLED display structures or manufacturing; 4) furthermore, the extraction enhancement offered by these methods/structures is generally still very limited and may be strongly wavelength and viewing‐angle sensitive,^24^, ^25^, ^26^, ^27^, ^28^, ^29^ not desirable for displays. Due to these difficulties, to date AMOLEDs hardly adopt any effective light out‐coupling techniques/structures for boosting efficiencies and power saving, although it is highly desired. Here we propose a general, highly effective, and scalable extraction‐enhancing OLED display pixel structure based on embedding the pixel OLED inside a 3D optically reflective concave structure selectively filled with a high‐index filler material. Optical simulation validates that ultimately high light extraction efficiency approaching ≈80% is achievable with such a 3D pixel configuration.

In the conventional AMOLED pixel structure shown in Figure 1a, other than electrical isolation and planarization, the tapering dielectric bank structure surrounding the emission aperture of the pixel OLED does not have particular optical functionality. The optical out‐coupling of the conventional pixel OLED is still dominated by its planar layered structure, and thus the optics (and poor light extraction) of the conventional pixel OLED is similar to that of a 2D planar OLED. However, by making good use of the existed slanted bank structure, one can convert the pixel OLED into a 3D optical structure bringing tremendous light extraction enhancement. Figure 1b illustrates how a 3D OLED pixel structure can be formed through modifying the conventional AMOLED pixel structure in Figure 1a; instead of only at the bottom surface, the bottom reflective electrode (e.g., Ag) is extended to the bank slopes surrounding the emission aperture; the actual emission aperture can be defined by an additional dielectric layer having an opening at the flat bottom surface and having a refractive index matching those of OLED active layers (e.g., SiNx often used in display industry); then after protection of the OLED with appropriate index‐matched passivation (e.g., index‐matched low‐temperature SiNx, if necessary), the pixel concave area is further filled with a high‐index filler material having a refractive index matching or exceeding those of OLED materials. In practice, the filler material (e.g., simply using the OLED active materials) may be selectively deposited into/around the concave area through tiny openings in a fine shadow mask (i.e., similar to patterned evaporation of OLED emitting layers, EMLs) or by other patterned deposition methods (e.g., jet printing); it may also simultaneously serve as the passivation/encapsulation (or other functionalities) for the AMOLED pixel. In a configuration like Figure 1b (called R‐bank + filler structure hereafter), the OLED emission zone is indeed embedded in an optically reflective concave structure (i.e., a reflective cup structure) with an optical index‐matching environment. With such a structure, it is aimed to first couple as much as possible internally generated photons into the high‐index filler region and then use the reflective concave structure to redirect otherwise confined light (i.e., light rays entering the filler with an initial internal angle θ_int_ larger than the total‐internal‐reflection (TIR) critical angle θ_c_ of the filler–air interface) for out‐coupling via one or multiple reflection by various surfaces, as illustrated by different light ray paths in Figure 1b. In addition to the R‐bank + filler structure, a pixel structure similar to the conventional structure except for being also selectively filled with the high‐index filler, i.e., the D‐bank + filler structure shown in Figure 1c, will also be studied for comparison.

In addition to enhancing light extraction, the proposed structure in Figure 1b is also expected to have the following features/merits: 1) the extraction mechanism is in principle wavelength insensitive, therefore generally good for all R/G/B colors; 2) the structure confines pixel emission mostly within the same pixel and thus shall minimize leakage/diffusion of a pixel emission to neighboring pixels and pixel blurring/mixing problems often seen in other OLED light extraction schemes; 3) there is no major change in optics of incident ambient light (i.e., no scattering/diffusive/diffractive reflection of incident ambient light) and therefore it shall not significantly alter the contrast characteristics of a conventional pixel; 4) requiring no major changes in the current top‐emitting AMOLED structure and no nanoscale fabrication, it holds promise for compatibility and feasibility for implementation.

Effects of the proposed structure on optical out‐coupling and overall optical properties, such as the spectral and angular dependence of emission, are quantitatively analyzed with optical simulation. Based on the proposed structure in Figure 1b, the pixel structure and corresponding dimension/feature definitions used for optical simulation are shown in Figure 1d, including the bank height *H*, the bank angle θ_B_, the bottom width *W*_1_ of the concave structure, the spacing *d* between the actual emission area and the bank edge, and the subpixel pitch *p* = *W*_1_ + 2*W*_2_ + 2*W*_3_. Assuming *H* of 2 µm and θ_B_ of 30° typical for real AMOLEDs, other representative subpixel dimensions used for simulation in this work are *W*_1_ = 13 µm, *d* = 1 µm, 2*W*_2_ ≈ 7 µm, 2*W*_3_ = 5 µm, and *p* = 25 µm, to mimic the situation in the real 500 ppi (pixel per inch) high‐resolution full‐color AMOLED display (having *a* ≈ 50 µm pixel pitch and 2 × 2 25 µm square subpixels). Values of *W*_1_, *H*, and θ_B_ are also varied to examine their influences on light extraction and other optical characteristics. The OLED active layer stacks are assumed to have the general structure of thick reflective Ag bottom electrode (150 nm)/hole‐transport layer‐HTL (*y* nm)/EML (10 nm)/electron‐transport layer—ETL (*x* nm)/(semi‐)transparent top electrode (either 20 nm Ag or 100 nm ITO), as shown in insets of Figure 1a–c. Actual optical constants [*n*(λ), *k*(λ)] of organic layers, Ag, and ITO were used in optical simulation (see Figure S2b, Supporting Information). For simplicity of simulation, the optical properties (refractive index *n*) of the typical host material 4,4′‐bis(carbazol‐9‐yl)biphenyl (CBP) (*n* ≈ 1.81 at 520 nm)^26^ are assumed for all the HTL, EML, ETL, and filler materials. Depending on the top (semi‐)transparent electrode adopted (thin Ag or ITO) and the bank optical properties (the R‐bank or conventional D‐bank with or without reflective Ag coating on the bank slope, respectively), four different structures: thin Ag + R‐bank, thin Ag + D‐bank, ITO + R‐bank, and ITO + D‐bank are studied.

The proposed OLED pixel contains structures of very different dimensional scales, i.e., nm‐scale structures that are smaller than wavelengths (e.g., thickness of the OLED active layers) and µm‐scale structures that are significantly larger than wavelengths (e.g., pixel size, bank height, filler thickness, etc.). Dealing the multiscale optical problem with fully electromagnetic wave optics is extremely challenging, if not impossible, since it would require huge computational resources and be extremely time consuming for dealing large‐scale portions of the structures; on the other hand, using fully geometrical optics/ray optics eases the computation issues but significantly sacrifices accuracy for small‐scale portions. Thus here optical properties of the proposed multiscale structure are analyzed with a multiscale optical simulation. It combines the analytical electromagnetic wave‐ and dipole‐based power dissipation model that is good for dealing detailed emission properties from nm‐scale layered structures,^8^, ^11^, ^12^, ^13^, ^32^ with the geometric optics simulation based on Monte Carlo ray tracing that is good for dealing optical properties of larger‐scale structures.^33^, ^34^, ^35^, ^36^, ^37^ With more details given in the Experimental Section and Figures S1–S3 in the Supporting Information, here the optical modeling/simulation methods are only outlined: 1) First, with the intrinsic emission spectrum and the emitting dipole orientation of the EML as inputs, the electromagnetic dipole model is used to calculate the coupling of the radiation generated in the OLED active region to the high‐index filler region (assumed semi‐infinite) as a function of the wavelength λ, polarization (s, p polarization), and initial internal angle θ_int_ in the filler. Such a distribution is then used to set up the light source for performing the ray‐tracing simulation in the macroscopic concave structure. 2) *R*_s_(λ, θ_AOI_) and *R*_p_(λ, θ_AOI_), the optical reflectance seen from the high‐index filler as a function of the polarization (*s*, *p*), λ, and angle of incidence (θ_AOI_) for each surface (bottom surface and bank slope) of the concave structure are also calculated with the electromagnetic wave theory. 3) With inputs from (1) and (2), the 3D polarization ray‐tracing simulation is then conducted to calculate extraction of the light from the high‐index filler region to air as a function of λ and external viewing angle θ.^36^, ^37^ The validity of the multiscale optical simulation relies on significantly diminished phase correlation (coherence) as light propagating in the µm‐thick filler, so that the finite‐thickness filler can be approximated as optically semi‐infinite (thus no strong interference effect from the filler layer) in calculating optical coupling into it and further light propagation in it can be treated with ray optics. This condition can be met if transparent high‐index filler materials yet with low degrees of coherence (e.g., nanocomposite materials etc.) are used for fillers. In addition, with the 3D R‐bank + filler pixel structure, the observed out‐coupled emission would mix directly out‐going emission and laterally propagated/redirected emission (of different initial internal angle θ_int_ and of different and much longer propagation paths); such redistribution and remixing shall also disturb/diminish phase correlation and interference effects with the finite‐thickness filler. We had also examined and compared simulation results of the simpler planar OLED structure (i.e., no bank structure) with different filler overcoating thicknesses by both the fully rigorous electromagnetic wave optics approach (assuming coherent filler) and the wave optics + ray optics approach (see the Experimental Section and Figure S19, Supporting Information, for details). For filler overcoating thickness above 1 µm, the wave + ray optics results agree well with the fully wave optics results within ±1%–±4.5% deviation percentage (as small as within ±1% for devices having ITO as transparent top electrode due to weaker optical interference in such device structures), indicating sufficient confidence and validation of the multiscale simulation approach adopted here for efficiency calculation.

For comparison and reference purposes, we first review characteristics of a conventional green top‐emitting OLED (dielectric bank, no high‐index filler) currently used in AMOLED displays, which has a typical structure: reflective Ag bottom electrode (150 nm)/HTL (*y* nm)/EML (10 nm)/ETL (*x* nm)/20 nm Ag plus 70 nm CBP capping or 100 nm ITO. Assuming the intrinsic emission spectrum and isotropic emitting dipole orientation (i.e., with a horizontal dipole ratio‐HR of 67%) of a typical green phosphorescent emitter Ir(ppy)_3_ for the EML,^26^, ^31^ Figures S4 and S5 in the Supporting Information show that the optimal light extraction efficiencies η_ext_ of ≈25–26% and ≈30% can be obtained with HTL/ETL of ≈200–205/60–65 nm for thin Ag and ITO devices, respectively. It is noted that higher extraction efficiencies are obtained with locating emitters at the second antinode position relative to the bottom reflective electrode (i.e., thicker HTL), mainly due to suppression of SPP loss to that metal contact.^13^, ^30^, ^31^ Such a larger HTL thickness indeed is the usual practice in current commercial AMOLED displays, not only for better light extraction but also for considerations of mass production yield and device reliability.^3^, ^4^, ^5^ Fortunately, current HTL materials could have sufficient conductivity/mobility to allow such a relatively large thickness without sacrificing the driving voltage. For the thin Ag device most used in current commercial AMOLED displays, since the device has a stronger microcavity effect, usually thinner HTL and ETL than optimal extraction conditions (to blueshift the microcavity resonant wavelength toward the intrinsic peak wavelength) are actually used to obtain more saturated colors and to reduce color shift versus viewing angles (see Figure S4, Supporting Information). As such, actual η_ext_ in commercial AMOLEDs could be lower than 20% (see Figure S4, Supporting Information; for instance, the conventional thin‐Ag top‐emitting OLED device having adjusted HTL/ETL thicknesses of 178/55 nm for color/viewing performance exhibits an η_ext_ of only ≈16%.)

We then discuss optical characteristics of the proposed extraction‐enhancing structures for a green subpixel, again assuming an isotropic green EML. **Figure** **2**a,b shows calculated η_filler_, the coupling efficiency of the radiation generated in the OLED active region to the high‐index filler (assumed semi‐infinite), as a function of the HTL and ETL thicknesses for both thin Ag and ITO devices, respectively. Optimal η_filler_ of up to ≈70% can be obtained for the thin Ag device with thicker HTL and ETL (e.g., both ≈200 nm), as SPPs to both metallic contacts are minimized. Meanwhile, significantly higher η_filler_ of up to ≈88% is seen for the ITO device with thick enough HTL (e.g., ≈200 nm), while the ETL thickness has minor effect since ETL, ITO, filler all have similar refractive indexes. The significantly higher η_filler_ of the ITO device is mainly associated with its less metal SPP, organic‐electrode WG, and absorption losses (compared to top thin Ag electrode) as to be detailed below. Since out‐coupled emission observed at an external viewing angle θ would have contribution from different initial internal angles θ_int_ through redirection by reflection, initial θ_int_‐dependent emission spectra and colors coupled into the filler need to be considered (see Figure S6, Supporting Information, for details) for obtaining better color performance in out‐coupled emission (e.g., more saturated colors at θ = 0° and smaller color shift vs viewing angles). Through detailed analyses of emission characteristics coupled into the filler, two thin Ag devices, **device 1** (thicker HTL/ETL of 200/200 nm) having highest η_filler_ (≈69.8%) and **device 2** (thinner HTL/ETL of 200/180 nm) having lower η_filler_ (≈68.6%) compromised with more acceptable colors at θ_int_ = 0° and smaller spectral/color shifts versus θ_int_ (see Figure S6, Supporting Information, for details) will be subjected to subsequent analyses. Meanwhile, the ITO device simultaneously having highest η_filler_ of ≈88% and small angle‐dependent spectra in the filler (**device 3**, with HTL/ETL thicknesses of 195/65 nm) will be further analyzed. Detailed optical modal distributions of radiation from these three devices in the EML and into the filler as a function of *k*_t_/*k*_o_ (*k*_t_ is the in‐plane wavevector in the device and *k*_o_ is the free‐space wavevector) with assuming a semi‐infinite filler are shown in Figure S7a–c in the Supporting Information. By examining detailed wave characteristics (*k*_t_/*k*_o_ range, polarization characteristics, field distribution, etc.) and by comparing mode distributions in EML/filler, air (radiation) modes, filler modes, organic‐electrode WG modes + loss, and SPP modes can be assigned and their fraction ratios are listed in Table S1 in the Supporting Information. With less SPP modes and significantly reduced organic‐electrode WG modes + loss, **device 3** exhibits a significantly higher air + filler mode ratio (corresponding to η_filler_) than **device 1/2** (87.8% vs 69.8%/68.6%). Air modes can be out‐coupled to air directly, while filler modes would otherwise be confined and waveguided in the filler without further extraction scheme (extraction of filler modes by the 3D pixel structure will be discussed later). Figure 2c–f shows the spectrally integrated emission intensities (s + p polarization; see Figure S7d–f in the Supporting Information) for separate p/s‐polarization emission patterns) and emission spectra coupled into the filler as a function of the initial internal angle θ_int_ for these three devices. There are significant distributions of emission over angles exceeding the filler–air TIR critical angle θ_c_ ≈ 33.5° (i.e., filler modes), which would not be out‐coupled if no effective extraction scheme is adopted. Variation of emission spectra versus θ_int_ is significant in **device 1** and is reduced in **device 2** as expected, while nearly negligible in **device 3** due to its very weak microcavity effect. Results of Figure 2c–f and Figure S7 in the Supporting Information are then used to set up the polarization‐, angle‐, and wavelength‐dependent light ray sources for performing the ray‐tracing simulation in the larger‐scale concave structure.

**Figure 2:**
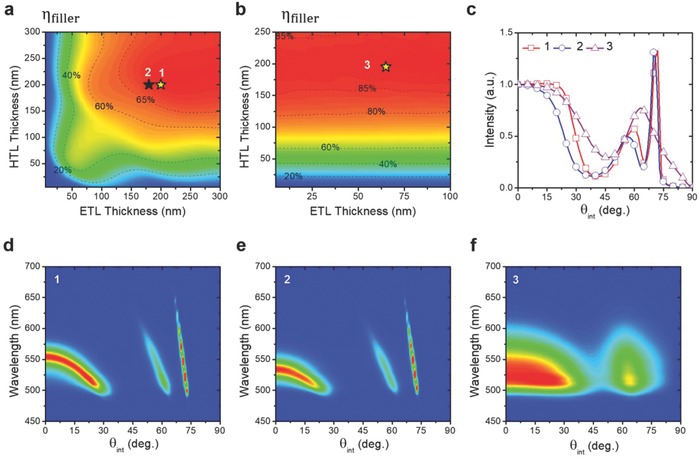
Pixel OLED emission coupled into the high‐index filler. a) Calculated η_filler_, the coupling efficiency of the radiation generated in the OLED active region to the high‐index filler, as a function of the HTL and ETL thicknesses for the thin Ag device: Ag (150 nm)/HTL (*y* nm)/EML (10 nm)/ETL (*x* nm)/Ag (20 nm)/CBP (semi‐infinite). b) Calculated η_filler_ for the ITO device: Ag (150 nm)/HTL (*y* nm)/EML (10 nm)/ETL (*x* nm)/ITO (100 nm)/CBP (semi‐infinite). c) Spectrally integrated radiation patterns coupled into the filler region for **devices 1**–**3**. d–f) Simulated angle‐dependent emission spectra coupled into the filler region for **devices 1**–**3**. Isotropic green emitters are assumed in the EML for all calculations.

*R*_s_(λ, θ_AOI_) and *R*_p_(λ, θ_AOI_) seen from the filler for each surface (bottom surface and bank slope) of the R‐bank/D‐bank structures containing **devices 1–3** are calculated with the analytical electromagnetic wave theory and corresponding layer structures, with calculation being detailed in the Experimental Section and calculated results being shown in Figure S8 in the Supporting Information. In summary, for all three device structures (**devices 1–3**), both bottom surfaces and R‐bank slopes generally show high reflection, while the ITO device exhibits the most homogeneous and highest reflection (>90%) over θ_AOI_, s/p polarizations, and λ. For the D‐bank slope surfaces, the ITO **device 3** exhibits consistently low reflection (<5%), except for θ_AOI_ exceeding the TIR critical angle. Meanwhile, the layer stacking of the thin Ag devices (**1** and **2**) on the D‐bank slope could result in certain reflection below the TIR critical angles, due to presence of the (more reflective) thin Ag top electrode.

With light ray sources and surface optical properties setup using results of previous sections, the 3D polarization ray‐tracing simulation is then conducted to calculate the light extraction efficiency from the filler to air, η_air_.^36^, ^37^ Calculated η_air_ for various devices in different pixel structures are listed in **Table** **1**. High η_air_ of ≈69–83% is obtained for green‐emitting **devices 1–3** with the R‐bank + filler structure (θ_B_ = 30°, *H* = 2 µm, *W*_1_ = 13 µm). Such high η_air_ can be achieved mainly because with appropriate bank angle θ_B_ (e.g., 30°), light rays with any initial θ_int_ > θ_c_ can all be successfully redirected into the <θ_c_ escape cone for out‐coupling (see Figure S9a, Supporting Information, for detailed illustration). Highest η_air_ of 83% obtained from the ITO + R‐bank device (**device 3** vs **devices 1** and **2**) is mainly due to its higher reflection (thus lower reflection loss) of all the surfaces (see Figure S8, Supporting Information) and also negligible absorption loss of the top transparent electrode (compared to thin Ag), since the light not directly escaping would undergo bouncing reflections between surfaces/interfaces before being lost or redirected for eventual out‐coupling. More than 75% of filler modes of **device 3** with the R‐bank + filler structure can be extracted (see Table S1, Supporting Information), while nearly half (46–48%) of filler modes are lost by absorption for **devices 1** and **2** with the R‐bank + filler structure. Without high‐reflection coating on the bank slope surface (i.e., D‐bank + filler structure), the ITO device exhibits low η_air_ of 30%. Interestingly, two thin Ag + D‐bank devices (**1, 2**) could still show moderate η_air_ of 48–52%, not surprising since thin Ag top electrode coated over bank slopes could provide certain reflection (see Figure S8, Supporting Information). With η_filling_ and η_air_, the overall optical extraction efficiencies η_ext_ (= η_filling_ × η_air_) for **devices 1–3** in R‐bank/D‐bank structures are obtained and summarized in Table 1. In the D‐bank + filler structure, not much enhancement in η_ext_ is seen for **device 3**; yet **devices 1** and **2** show more significant enhancement in η_ext_ (≈33–36% vs ≈16–24% of the color‐compromised conventional top‐emitting pixel device as detailed in Figures S4 and S5, Supporting Information) with its sufficient η_filling_ and η_air_. In contrast, all **devices 1–3** in the R‐bank + filler structure exhibit significantly high η_ext_ of 49.2%, 47.1%, and 72.6%, respectively (cf. the D‐bank + filler structure and conventional top‐emitting OLED). In particular, the ITO device in the R‐bank + filler structure gives extremely high η_ext_ of ≈73%, which is four to five times higher than that (≈16%) of current top‐emitting AMOLED pixels. Such high η_ext_ could reduce power consumption of an AMOLED by nearly a factor of five, which is significant for various mobile and power‐aware applications.

**Table 1:** Light coupling/extraction efficiencies under various structures/conditions

Devicea)	Bank	Color	HR	η_filler_ [%]	η_air_ [%]	η_ext_ [%]
1	D	Green	67	69.8	51.6	36.0
	R				70.6	49.2
2	D	Green	67	68.6	47.8	32.8
	R				68.7	47.1
3	D	Green	67	87.8	30.5	26.7
	R				82.6	72.6
3	D	Red	67	93.7	29.4	27.5
	R				85.9	80.5
3	D	Blue	67	83.5	30.1	25.2
	R				80.2	66.9
1	R	Green	100	70.2	77.7	54.5
3	R	Green	100	88.4	86.2	76.2

^a)^ θ_B_ = 30°, *H* = 2 µm, *W*_1_ = 13 µm.

**Figure** **3**a–d depicts the calculated angle‐dependent emission intensities (cf. the Lambertian pattern) and emission spectra (cf. emitter's intrinsic emission spectrum) for **devices 1–3** in the R‐bank + filler structure (see Figure S10, Supporting Information, for results of the D‐bank + filler structure). Although **device 1** in the R‐bank + filler structure could give much higher η_ext_ than conventional top‐emitting OLED, it shows significant variation of emission spectra/colors versus viewing angles and also some stronger smaller‐angle features in the emission pattern, both indeed reflecting its initial θ_int_‐dependent emission spectra and intensity in the filler (Figure 2c,d and Figure S7, Supporting Information), the redirection of larger‐angle radiation toward smaller angles (e.g., peaks around θ_int_ ≈ 60° and 70° being redirected to θ_int_ ≈ 0° and 18°, respectively; see Figure S9a, Supporting Information), and its superposition onto original smaller‐angle radiation. **Device 2** in the R‐bank + filler structure exhibits more directed emission and yet reduced color shift versus angles (cf. **device 1**). Most importantly, the ITO device (**device 3**) exhibits an emission pattern closest to the Lambertian profile and also negligible color shift over angles, both being highly desired for high‐end display applications. Such nice viewing characteristics together with its extremely high η_ext_ makes the ITO top electrode + R‐bank + filler structure the ideal pixel structure and technology for high efficiency (low power consumption) and high image quality OLED displays.

**Figure 3:**
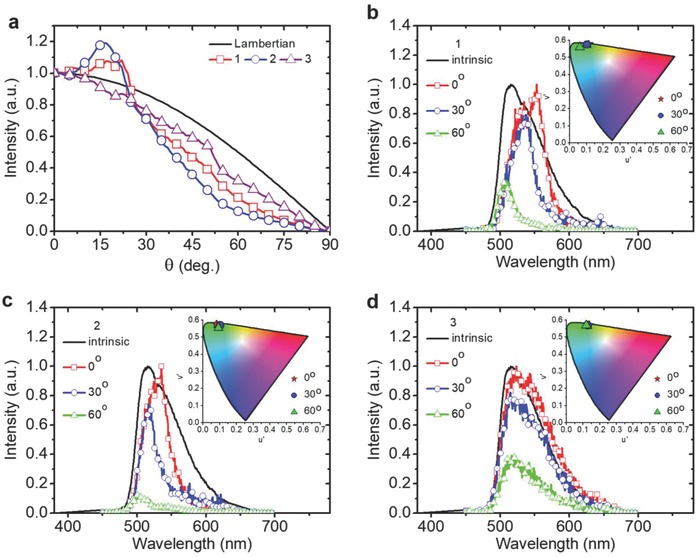
Out‐coupled pixel OLED emission for R‐bank + filler structures. *H* = 2 µm, θ_B_ = 30°, *W*_1_ = 13 µm, *d* = 1 µm, 2*W*_2_ ≈ 7 µm, 2*W*_3_ ≈ 5 µm, *p* = 25 µm are assumed. a) Viewing‐angle dependent (spectrally integrated) out‐coupled emission intensity for **devices 1**–**3** in the R‐bank + filler structure. The Lambertian pattern is also shown for comparison. b–d) Viewing‐angle dependent out‐coupled emission spectra for **devices 1**–**3** in the R‐bank + filler structure. The intrinsic emission spectrum of the isotropic green emitter in EML is also shown for comparison.

We further examine the influences of different pixel structure parameters, such as the bank angle θ_B_, the bank height *H*, and the pixel size on light extraction (mainly R‐bank results here; see Figure S13, Supporting Information, for D‐bank results). **Figure** **4**a shows η_ext_ as a function of θ_B_ for **devices 1–3** in the R‐bank + filler structure (with other parameters fixed). Clearly, a θ_B_ of 10°–40° is most effective and optimal for re‐directing larger‐angle light in the filler for out‐coupling, while the gain is less optimized at larger or smaller θ_B_. For **device 3**, highest η_ext_ of 74% is obtained at θ_B_ ≈ 20°. Generally speaking, with an appropriate θ_B_ (e.g., 10°–30°), light rays with initial θ_int_ > θ_c_ can be effectively redirected into <θ_c_ for out‐coupling with least numbers of reflection in the structure (see Figure S9b, Supporting Information, for detailed illustrations of various light ray paths in the structures with different θ_B_). Figure 4b shows η_ext_ as a function of *H*, which shows an increasing trend versus *H*. For **device 3**, an η_ext_ of >75–76% can be obtained with *H* ≥ 4 µm. It is quite understandable since a larger bank height (thus larger filler depth and larger H/*W*_1_ aspect ratio) would reduce the number of bouncing reflections and corresponding optical loss in the bank + filler structure. Similarly, Figure 4c shows η_ext_ as a function of the pixel size (by varying emission aperture *W*_1_, corresponding to different display ppi resolutions). The smaller the pixel (*W*_1_, i.e., higher ppi), the more effective the R‐bank + filler structure for enhancing light extraction. It is a highly favorable advantage of the current extraction scheme for future development of even higher resolution AMOLEDs. Finally, When combining all the favorable conditions (e.g., θ_B_ ≈ 20°, *W*_1_ ≈ 6.5 µm, *H* ≥ 4 µm), **device 3** in the R‐bank + filler structure can give an η_ext_ as high as ≈78%. One notices that the conditions for optimal η_ext_ (larger *H*, smaller θ_B_) may lead to larger bank widths, not good for fill factors of emission areas at high pixel densities. Fortunately, larger fill factors can be achieved with less optimal efficiency conditions (e.g., larger θ_B_ of 30°–35°, smaller *H* of <2 µm), and yet still keep most of η_ext_ (only slight η_ext_ tradeoff within a few %, see Figure 4a,b) In addition, as the pixel size (e.g., *W*_1_) shrinks, the required *H* for optimal efficiency also scales down proportionally (see Figure 4d for **device 3** results), since η_ext_ is determined by the *H*/*W*_1_ aspect ratio, not the absolute H value (see inset of Figure 4d). Thus indeed for *W*_1_ of <10 µm, required *H* could be of ≤1 µm, minimizing impacts on fill factors. Alternatively, η_ext_ can also be correlated with the fill factor (e.g., varying *W*_1_ and *W*_1_ + 2*W*_2_ but setting subpixel pitches *p* = *W*_1_ + 2*W*_2_ + 2*W*_3_ at fixed values. Figure 4e,f shows η_ext_ as a function of the fill factor for **devices 1** and **2** and **device 3** with *p* = 25, 30, 35 µm, and *H* = 2 µm, θ_B_ = 30°. Apparently, for a same *p*, η_ext_ drops as the filler factor increases, but the drop is less for smaller *p* (higher pixel densities); for a same fill factor, η_ext_ increases as *p* decreases (higher pixel densities). All these of course again correlate with the actual dimensions of *W*_1_ and *W*_1_ + 2*W*_2_.

**Figure 4:**
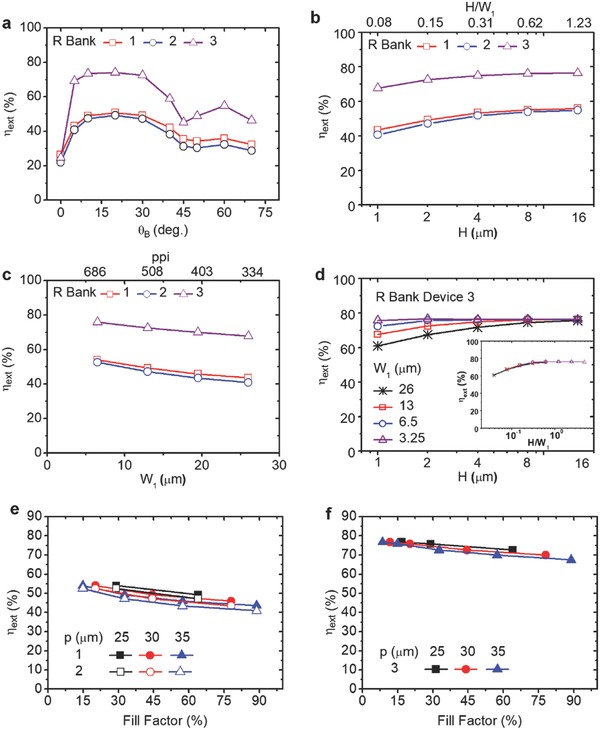
Influences of structures on out‐coupling efficiency in the R‐bank + filler structure. a) Calculated η_ext_ as a function of the bank angle θ_B_ for **devices 1**–**3**, with *W*_1_ = 13 µm, *H* = 2 µm. b) Calculated η_ext_ as a function of the bank height *H* for **devices 1**–**3**, with *W*_1_ = 13 µm, θ_B_ = 30°. c) Calculated η_ext_ as a function of the bottom width *W*_1_ for **devices 1**–**3**, with H = 2 µm, θ_B_ = 30°. Assume isotropic green emitters in all devices. d) Calculated η_ext_ as a function of the bank height *H* for **device 3** having different *W*_1_ of 26, 13, 6.5, and 3.25 µm, and θ_B_ = 30°. The inset of (d) depicts η_ext_ as a function of *H*/*W*_1_ for **device 3** of different *W*_1_, to show that η_ext_ is universally governed by the *H*/*W*_1_ aspect ratio, not the absolute *H* value. e,f) Calculated η_ext_ as a function of the fill factor (i.e., with varied *W*_1_ and *W*_1_ + 2*W*_2_) for **devices 1** and **2** and **device 3**, with subpixel pitches (*p* = *W*_1_ + 2*W*_2_ + 2*W*_3_) fixed at 25, 30, and 35 µm, and with *H* = 2 µm, θ_B_ = 30°.

To examine whether the light extraction scheme can be extended to red and blue subpixels of full‐color AMOLEDs as well, similar analyses were conducted for red and blue subpixels in the ITO + R‐bank + filler structure (**device 3**‐like, with *W*_1_ = 13 µm, *H* = 2 µm, θ_B_ = 30°), using hypothetical red and blue emission spectra for the EML (by shifting the intrinsic emission spectrum to 620 and 470 nm, respectively, see Figures S14 and S15, Supporting Information, for details of simulation and results). Optimized η_filler_, η_air,_ and η_ext_ obtained for red and blue devices are (93.7%, 85.9%, 80.5%) and (83.5%, 80.2%, 66.9%), respectively (Table 1 and Figures S14 and S15, Supporting Information). Similarly high η_ext_ of 67–81% achievable for all red, green, and blue subpixels clearly demonstrates that the light extraction scheme and mechanism here are not sensitive to emission wavelengths and are generally applicable to different colors.

So far, emitters in the OLEDs are assumed isotropic with a horizontal dipole ratio HR of 67%. One of recent trends is to develop OLED emitting materials with more preferentially horizontal emitting dipoles (i.e., with >67% horizontal dipole ratios),^11^, ^12^, ^38^, ^39^, ^40^, ^41^ so that the radiation patterns inside the OLED are more favorable for direct out‐coupling, the loss or trapping to waveguided/SPP modes is reduced, and the light extraction from a planar OLED can be enhanced even without adopting any optical out‐coupling structures. For instance, for the conventional top‐emitting green device (thin Ag or ITO), the light extraction efficiency η_ext_ compromised with viewing/color performance can be enhanced to some degree from 16–24% (for isotropic emitters) to ≈34% by adopting purely horizontal dipole emitters with HR = 100% (see Figures S16 and S17, Supporting Information, for details). However, even with perfectly horizontal emitting dipoles, η_ext_ for conventional top‐emitting OLEDs is still far from ideal, not to mention that highly horizontal dipole emitters simultaneously with high emission efficiency are yet to be realized. When replacing the isotropic green emitter with the ideal 100% horizontal dipole emitters in the ITO + R‐bank + filler structures (with representative *W*_1_ = 13 µm, *H* = 2 µm, θ_B_ = 30°), the optimized η_filler_, η_air,_ and η_ext_ only slightly rise to 88.4%, 86.2%, and 76.2%, respectively, compared to 87.8%, 82.6%, and 72.6% of the isotropic case (see Table 1 and Figure S18, Supporting Information). Such results clearly indicate the R‐bank + filler structure is very effective for extracting most of the OLED emission regardless of dipole orientations, not relying on ideal horizontal dipole emitters yet to be realized.

Finally, although substantial works are still required to realize the proposed pixel structures, possible ways, and requirements for implementation and impacts of implementation variation/deviation are further discussed. The most critical step for implementation, i.e., selective/patterned deposition of the high‐index filler into or around the concave region, is challenging but maybe not so inconceivable or far away in view of current available/known technologies. For instance, some current OLED thin‐film encapsulation technology already adopts inkjet printing to deposit (rather thick) polymer precursors in the inorganic/organic multilayer thin‐film encapsulation stacks.^42^, ^43^ Such technique may be appropriately adapted for selective/patterned deposition of the high‐index filler subsequent to deposition of inorganic passivation/protection layers (such as the common index‐matched low‐temperature SiNx) over the OLED top electrode, a process that is not so different from current available technologies and may simultaneously provide patterning, encapsulation, and light extraction functionalities. In addition, modulating surface wetting properties with appropriate surface treatment may also give self‐alignment capability to ensure alignment accuracy of the inkjet printing process.^44^ Similar to inkjet printing, vapor‐phase jet printing may also be used for patterned deposition of the high‐index (organic) filler materials.^45^ As another instance, very high‐resolution patterning of OLED evaporation layers (>1000 ppi) has been recently demonstrated by using planar evaporation sources, very high‐resolution shadow masks, and by bringing substrates into close proximity of sources.^46^ By evaporation through shadow‐mask openings smaller than or similar to the concave area and by utilizing the shadowing effects of evaporation, patterned high‐index filler layers with approximate truncated pyramid shape (i.e., tapered layer thickness around the patterned layer edge, as demonstrated by some previous works^46^, ^47^) for fitting in the concave area shall be feasible. In addition, such proximity evaporation shall substantially raise the material utilization efficiency (thus reduce material cost), not to mention that high‐index filler need not be costly active OLED materials.

With the above mentioned techniques, depending on alignment accuracy, inkjet drop sizes/amounts, ink or surface/interface properties, shadowing profiles of evaporation, etc., actually implemented structures (particularly the selective/patterned filler) somewhat may still have deviation from that shown in Figure 1b, resulting in varied structures (**reference**, **A**, **B**, **C**, **D**) schematically shown in **Figure** **5**a. Structures **A** and **B** represent the cases in which the filler mainly dwells in the concave area (*W*_1_ + 2*W*_2_) but has varied height distributions (thicker or thinner at the center); structures **C** and **D** represent the cases in which the filler layer extends over the bank top (with bottom reflector also extended to the bank top), either centering around the concave area (extension Δ*W* on each side) or shifted/misaligned by Δ*X* (Δ*W* − ΔX, Δ*W* + ΔX on each side). To evaluate their impacts, η_ext_s of these structures (still *W*_1_ = 13 µm, θ_B_ = 30°, *H* = 2 µm) with **device 3** OLED stacks (green, isotropic) embedded are calculated and summarized in **Table** **2**. All structures can retain η_ext_s similar to that of Figure 1b (**reference**) structure (even slightly higher for some cases), except for slight reduction for structures **C** and **D** associated with slightly more reflection loss in some longer ray propagation paths before out‐coupling (see Figure 5b). With the filler layer extended to the bank top (thus not completely confined within the bank), some light rays could be guided by the filler to the bank top and be out‐coupled there, encountering longer propagation paths and possibly more reflection loss (see Figure 5b). Nevertheless, as long as the filler layer is patterned (not continuous and connected to neighboring pixels), most of radiation entering the filler would still be eventually out‐coupled (although the out‐coupling mechanism may vary). Overall, results of Figure 5 and Table 2 indicate that the proposed light extraction configuration/method is rather tolerant of structure variations, substantially relaxing requirements for perfect or tight control of the filler geometry or topography. Also, as long as the filler layer is patterned, diffusion/leakage of a pixel emission to neighboring pixels (and even out‐coupling by neighboring pixels) and thus pixel image blurring usually associated with such phenomena would be minimized. Finally, even with a surface topography being not totally flat (i.e., with surface corrugation) as shown in Figure 5a, its effect to scattering/reflection of incident ambient light would not be any worse (thus not a bigger concern than the conventional OLED display structure shown in Figure 1a, which with no filler in bank structures, indeed also has rather large surface corrugation). Even if there is scattered/stray light from edges of pixel structures (in Figure 5a), it can be remedied by blocking with appropriate black matrices as in the usual display practice.

**Figure 5:**
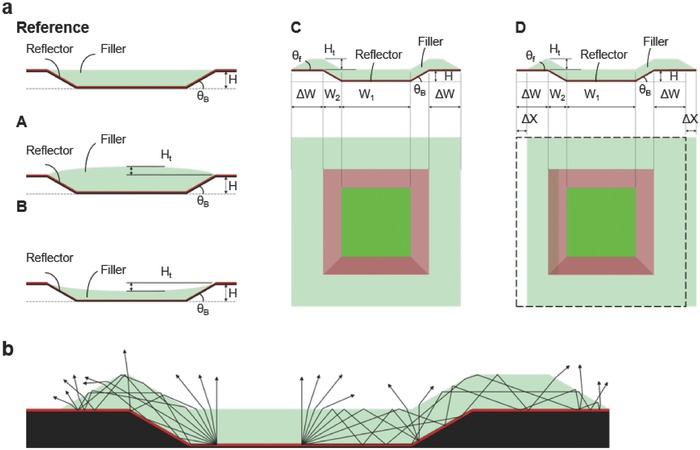
Possible variations in implemented pixel/device configurations. a) Schematic diagrams of various structures. The **reference** structure is like that in Figure 1b; structures **A** and **B** represent the cases in which the filler mainly dwells in the concave area (*W*_1_ + 2*W*_2_) but has varied height distributions (thicker or thinner at the center by *H*_t_); structures **C** and **D** represent the cases in which the filler layer and the bottom reflector extend over the bank top with a maximal thickness *H*_t_ over the bank top, either centering around the concave area (extension Δ*W* on each side) or shifted/misaligned by Δ*X* (extension Δ*W* − Δ*X*, Δ*W* + Δ*X* on each side). θ_f_ is the tapering angle of the filler layer relative to the bank top around the edge of the filler pattern. b) Schematic illustration of how light rays originating from different locations with different initial internal angle θ_int_ in the filler propagate in the reflective bank + filler structure **D** and are out‐coupled. For simplicity, some structure details, such as additional dielectric, OLED stacks, possible index‐matched passivation layer above OLED top electrode, etc., are omitted and not explicitly shown in these schematic diagrams.

**Table 2:** Light extraction efficiencies for different structure variations in Figure 5a

Structurea)	Δ*W* [µm]	Δ*X* [µm]	*H*_t_ [µm]	η_ext_ [%]
Reference	0	0	0	72.6
A	0	0	0.25	71.9
	0	0	0.5	71.5
B	0	0	− 0.25	73.1
	0	0	− 0.5	73.0
C	6	0	0.5	68.3
	6	0	2	66.6
D	6	0.5	0.5	68.3
	6	0.5	2	66.7
	6	2	0.5	68.3
	6	2	2	66.8

^a)^ **Device 3** (OLED stack including bottom reflective electrode), Color = Green, HR = 67%, R Bank, θ_B_ = 30°, *H* = 2 µm, *W*_1_ = 13 µm, θ_f_ = 30°.

In summary, we report a highly effective extraction‐enhancing pixel structure design for OLED displays, which is based on embedding the top‐emitting pixel OLED inside a 3D reflective concave structure selectively covered with a high‐index filler material. Such a structure is able to couple as much as possible internally generated photons into the filler region and then use the reflective concave structure and the patterned filler to redirect otherwise confined light for out‐coupling. Comprehensive multiscale optical simulation was conducted on light extraction of such structures; simulation validates that ultimately high light extraction efficiency approaching ≈80% and excellent viewing characteristics are simultaneously achievable with optimized structures using highly transparent top electrodes. This scheme is general and scalable; it is not wavelength sensitive and thus generally applicable to all red, green, and blue pixel OLEDs in full‐color displays. The extraction efficiency increases in shrinking the pixel size and thus is particularly advantageous for very high‐resolution OLED display. In addition, the high efficiency does not rely on ideal horizontal dipole emitters that are not yet realized. It is also quite tolerant of structure variations and imperfections, as long as the filler layers are patterned. Although implementation of such structures require further development and innovation in advanced fabrication techniques and materials, nevertheless results of this work shall have an important impact in providing a clear vision and direction for and shedding light on solving the long‐lasting light extraction problem and development of future generations of advanced OLED displays. General concepts/designs reported in our work are also believed to be generally applicable to all types of self‐emissive displays/devices, e.g., quantum‐dot LED, micro‐LED, and perovskite LED displays etc., in the format of rigid, flexible, to even rollable displays.

## Experimental Section

Optical properties of the proposed structure were analyzed with a multiscale optical simulation, which combines the analytical electromagnetic wave‐ and dipole‐based power dissipation model (see the Supporting Information for more details) for dealing detailed emission properties from nm‐scale layered structures, with the geometric optics simulation based on 3D polarization Monte Carlo ray tracing for dealing optical properties of larger‐scale structures (see the Supporting Information for more details). The complete simulation in general consisted of three major steps.

First, with the OLED layer structure and the intrinsic emission spectrum and the emitting dipole orientation of the EML as inputs, the electromagnetic dipole model was used to calculate the coupling of the radiation generated in the OLED active region to the high‐index filler region (assumed semi‐infinite) or air as a function of the wavelength λ, polarization (s, p polarization), and initial internal angle θ_int_ in the filler (or external viewing angle θ in air). From such calculation, the light coupling efficiency from the OLED active region to the high‐index filler (i.e., η_filler_) and the emission intensity distribution (as a function of wavelength, polarization, initial internal angle θ_int_) in the filler were obtained. The initial emission intensity distribution in the filler thus obtained was then used to set up the light ray sources for performing the ray‐tracing simulation in the larger‐scale concave structure. The stack of OLED active layers were assumed to have the general structure of thick reflective Ag bottom electrode (150 nm)/HTL (*y* nm)/EML (10 nm)/ETL (*x* nm)/(semi‐)transparent top electrode (either 20 nm Ag or 100 nm ITO). Actual optical constants [*n*(λ), *k*(λ)] of organic layers, Ag, and ITO were used in optical simulation (see Figure S2b, Supporting Information). For simplicity of simulation, the optical properties (refractive index n) of the typical host material CBP (*n* ≈ 1.81 at 520 nm) were assumed for all the HTL, EML, ETL, and filler materials.

Second, *R*_s_(λ, θ_AOI_) and *R*_p_(λ, θ_AOI_), the optical reflectance seen from the high‐index filler as a function of the polarization (s, p), λ, and angle of incidence (θ_AOI_) for each surface (bottom surface and bank slope) of the concave structure were also calculated with the electromagnetic wave theory. For the bottom surface, the layer structures of Ag bottom electrode (150 nm)/CBP (of corresponding thickness)/(semi‐)transparent top electrode (either 20 nm Ag or 100 nm ITO) for **devices 1–3** were used to calculate the optical reflection and transmission seen from the high‐index filler. For simplicity, the additional dielectric layer that may be needed to define the actual emission aperture in implementation of the R‐bank structure was omitted in most of simulation and discussion, since simulation shows very similar results with or without this additional dielectric layer (see Figures S11 and S12 and Table S2, Supporting Information). Thus for calculating reflection and transmission of the R‐bank slope surface, the layer structures similar to the bottom surface, except for all layer thicknesses being multiplied by cosθ_B_ (θ_B_ is the bank angle) for taking into account the effect of oblique‐angle deposition of material layers onto the bank slope [i.e., Ag bottom electrode (150 × cosθ_B_ nm)/CBP (of corresponding thickness × cosθ_B_)/(semi‐)transparent top electrode (either 20 × cosθ_B_ nm Ag or 100 × cosθ_B_ nm ITO) for **devices 1–3**], were used to calculate the optical reflection and transmission of the bank slope surface seen from the high‐index filler. Meanwhile, the layer structures of semi‐infinite D‐bank/CBP (of corresponding thickness × cosθ_B_)/(semi‐)transparent top electrode (either 20 × cosθ_B_ nm Ag or 100 × cosθ_B_ nm ITO) for **devices 1–3** were used to calculate the optical reflection and transmission of the D‐bank slope surface seen from high‐index filler. *n* ≈ 1.5, typical for transparent photoresists used to form the bank structure, was assumed for the dielectric bank material.

Third, with setting up ray sources, surface optical properties and the geometric structure of the concave structure, the 3D polarization Monte Carlo ray‐tracing simulation with the LightTools software (Synopsys, Inc.) was then conducted to calculate extraction of the light from the high‐index filler region with an initial ray quantity of 200 million. Eventually the overall light extraction efficiency from the high‐index filler to air (i.e., η_air_) and the overall out‐coupled far‐field emission intensity in air as a function the wavelength λ and the external viewing angle θ. The overall light extraction efficiency from the OLED active region to air (i.e., η_ext_) can then be obtained by η_ext_ = η_filler_ × η_air_. The intensity distributions and the efficiency in air were collected by an enclosed sphere with an infinite radius. To avoid collection of the leakage modes and emission toward lateral sides of the structure and to ensure only collection of emission toward the forward direction, the lateral side surfaces and the bottom surfaces of the structure were set to be absorptive in the simulation model. The angular spectra were collected by apertures of 1° full cone angle at corresponding angles (e.g., 0°, 30°, 60°, etc.).

To examine the validity of the multiscale optical simulation, e.g., to what extent the thick filler layer can be treated as semi‐infinite and light propagation in it can be treated with ray optics, testing and comparison simulation were conducted on a simpler planar structure (i.e., no bank structure) with **devices 1–3** OLED stack (green, isotropic) but different filler overcoating thicknesses (see Figure S19, Supporting Information, for the configuration) by: i) the fully rigorous electromagnetic wave optics approach that deals the OLED stack and the filler overcoating as a whole by wave optics and ii) the wave optics + ray optics approach that first uses the wave optics to calculate coupling of OLED internal emission into the filler layer (assumed semi‐infinite) and then uses the ray optics to further calculate out‐coupling from the filler to air as described in the Experimental Section (except no bank slopes need to be considered here). As shown in Figure S19 in the Supporting Information, η_ext_s calculated by both methods agree well within a ±1%–±4.5% deviation percentage (even as small as within ±1% for devices having ITO as transparent top electrode) for the filler overcoating thickness of >1 µm, indicating sufficient confidence in calculation of optical out‐coupling efficiency in this work (which mainly deal with filler thicknesses of ≥1 µm).

## Conflict of Interest

The authors declare no conflict of interest.

## Supporting information

Supplementary
