# Supplementary material for: A Vision toward Ultimate Optical Out‐Coupling for Organic Light‐Emitting Diode Displays: 3D Pixel Configuration
Source: Adv Sci (Weinh). 2018 Aug 29;5(10):1800467. doi: 10.1002/advs.201800467 (PMC6193169; doi:10.1002/advs.201800467)
Supplement: Supplementary file 1 — Supplementary [file ADVS-5-1800467-s001.pdf]

Copyright WILEY-VCH Verlag GmbH & Co. KGaA, 69469 Weinheim, Germany, 2018.

Supporting Information

for *Adv. Sci.,* DOI: 10.1002/advs.201800467

A Vision toward Ultimate Optical Out-Coupling for Organic Light-Emitting Diode Displays: 3D Pixel Configuration

*Yi-Jiun Chen, Wei-Kai Lee, Yi-Ting Chen, Chun-Yu Lin, Sheng-Wen Wen, Min Jiao, Guo-Dong Su, Hoang Yan Lin, Robert J. Visser, Byungsung Leo Kwak, Chung-Chia Chen, Wan-Yu Lin, Steve Wang, Chorng-Ping Chang, and Chung-Chih Wu**

Supporting Information

A Vision towards Ultimate Optical Out-coupling of Organic Light-emitting Diode Displays: Three-Dimensional Pixel Configuration

*Yi-Jiun Chen‡, Wei-Kai Lee‡, Yi-Ting Chen, Chun-Yu Lin, Sheng-Wen Wen, Min Jiao, Guo-Dong Su, Hoang Yan Lin, Robert J. Visser, B. Leo Kwak, Chung-Chia Chen, Wan-Yu Lin, Steve Wang, Chorng-Ping Chang, Chung-Chih Wu**

Yi-Jiun Chen, Wei-Kai Lee, Yi-Ting Chen, Chun-Yu Lin, Sheng-Wen Wen, Min Jiao, Prof. Guo-Dong Su, Prof. Hoang Yan Lin, Prof. Chung-Chih Wu

Graduate Institute of Photonics and Optoelectronics, Graduate Institute of Electronics Engineering, Department of Electrical Engineering, National Taiwan University, Taipei 106, Taiwan

Email: wuCC@ntu.edu.tw

Robert J. Visser, B. Leo Kwak, Chung-Chia Chen, Wan-Yu Lin, Steve Wang, Chorng-Ping Chang

Applied Materials, Inc., California 95052, United States of America

‡ These authors contributed equally to this work.

1. More details of optical simulation methods

Since the proposed OLED pixel contains structures of very different dimensional scales, i.e., nm-scale structures that are smaller than wavelengths (e.g., thicknesses of the OLED active layers) and μm-scale structures that are significantly larger than wavelengths (e.g., pixel size, bank height, filler thickness etc.), optical properties of the proposed structure are analyzed with a multi-scale optical simulation, as schematically illustrated in **Figure S1**. It combines the rigorous and analytical electromagnetic wave-and dipole-based power dissipation model that is good for dealing detailed emission properties from nm-scale layered structures[1-5], with the geometric optics simulation based on Monte Carlo ray tracing that is good for dealing larger-scale structures[6-10]. The optical modeling/simulation for the proposed structures then contains three steps, as shown in the flow chart of **Figure S2**: (1) **step 1**: calculate the emission properties coupled from the OLED active region into the filler region with the analytical electromagnetic model for use in setting up ray sources in subsequent ray-tracing simulation; (2) **step 2**: calculate optical properties (reflectance, transmittance) for various surfaces of the proposed structures with the analytical electromagnetic model for use in subsequent ray-tracing simulation; (3) **step 3**: with inputs from **step 1** and **step 2**, setting up and performing the 3D polarization ray-tracing simulation of the proposed structure.

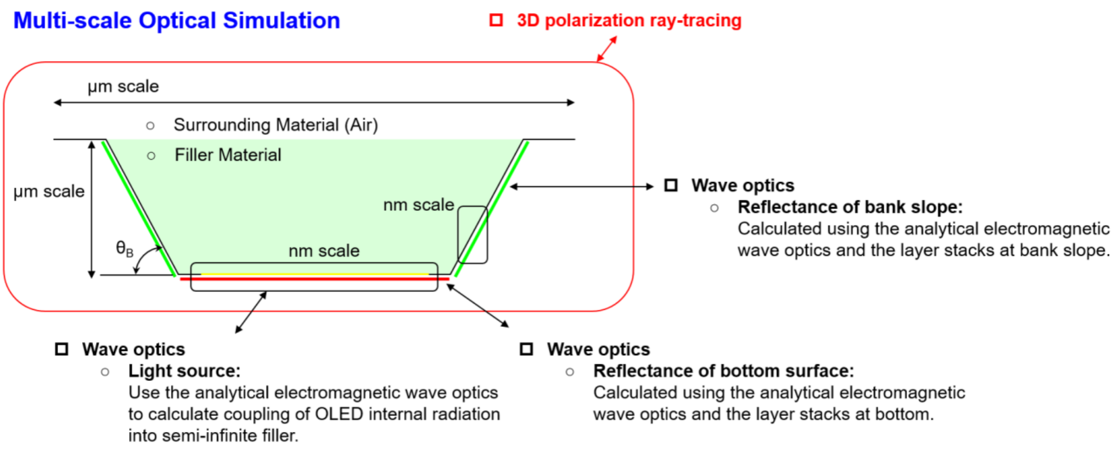

Figure S1. The multi-scale optical simulation approach used in this work.

a

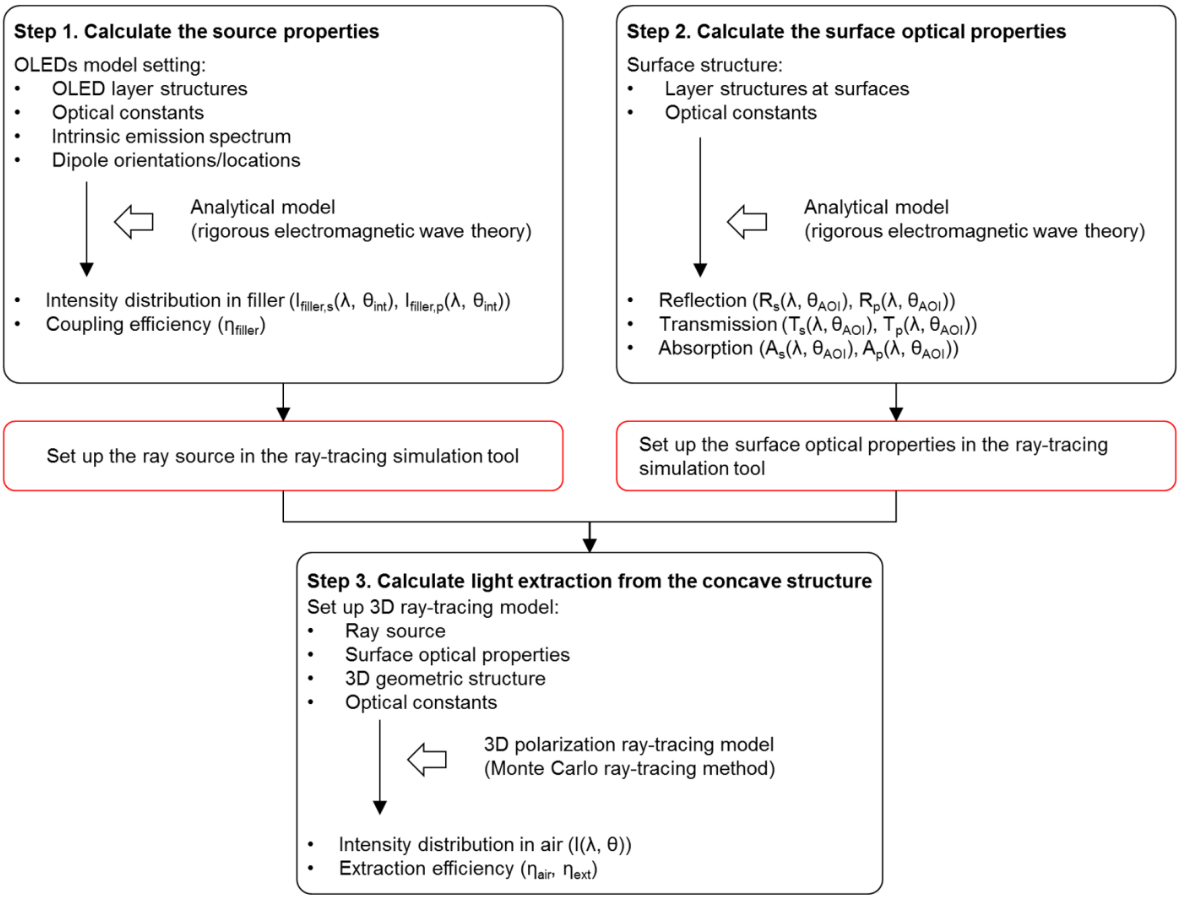

b

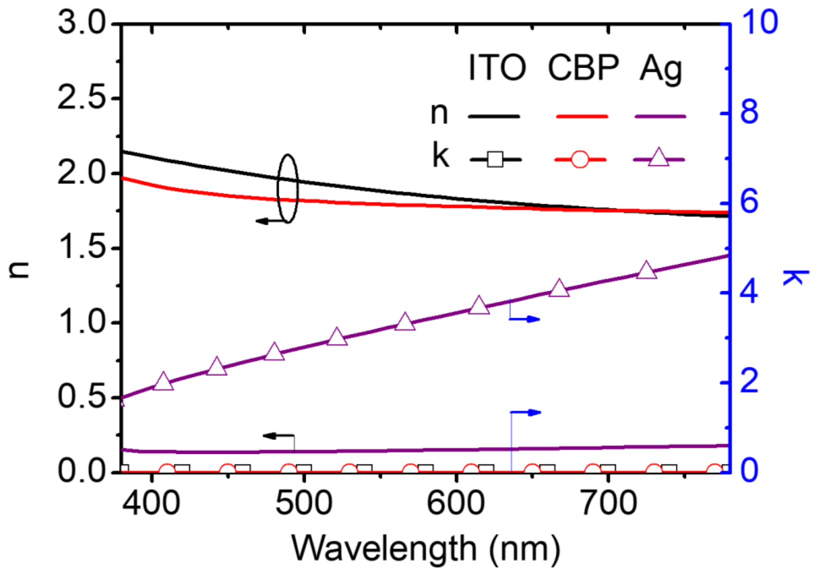

Figure S2. (a) The flow chart for the optical simulation in this work. (b) Optical constants n(λ) and k(λ) of CBP, Ag, and ITO used in the optical simulation.

Step 1:

First of all, with the OLED layer stacking structure, and the intrinsic emission spectrum and the emitting dipole orientation of the EML as inputs, the analytical electromagnetic dipole model is used to calculate the coupling of the radiation generated in the OLED active region to the high-index filler region (by assuming it is semi-infinite) as a function of the wavelength λ, the polarization (s, p), and the initial internal angle θ_{int} in the filler. From such calculation, the light coupling efficiency from the OLED active region to the high-index filler (i.e., η_{filler}) and the s-polarization/p-polarization/total emission patterns in the filler are obtained. Such radiation intensity distributions in the filler $I_s(\lambda, \theta_{int})$, $I_p(\lambda, \theta_{int})$ as a function of the wavelength λ, the polarization (s, p), and the internal angle θ_{int} are then used to set up ray sources in the filler for performing the ray-tracing simulation in the larger-scale concave structure (**step 3**).

The analytical electromagnetic dipole model (the analytical model) used in **step 1** for calculating coupling of radiation generated in the OLED active region to the high-index filler region or air (for conventional top-emitting OLED) adopts a classical approach based on the equivalence between the molecular emission through electronic dipole transitions and the electromagnetic radiation from a classical electrical dipole antenna[1-5]. Such a model can take into account coupling of OLED emission into SPP and waveguided modes and the loss due to the electrodes. Using the plane-wave expansion of the dipole radiation field, the full-vectorial electromagnetic fields generated by a radiation dipole embedded in a layered structure can be calculated, from which the distribution of the radiation power into different plane-wave modes and the far-field radiation (e.g., coupling of OLED internal radiation into the semi-infinite filler region in proposed OLED structures or into air for conventional top-emitting OLEDs) related to emission characteristics of the OLED stack can be obtained. In the plane-wave expansion calculation, each plane-wave mode can be characterized by an in-plane wave vector k_t, where k_t is the component of the wave vector parallel to the planar layer surface/interface. Emission characteristics of an OLED are calculated by assuming that the emitting layer (EML) contains an ensemble of mutually incoherent dipole radiators with distributions in dipole orientations (e.g., a random isotropic distribution or some anisotropic distributions of the emitting dipoles), locations, and frequencies (e.g., the full spectral distribution from the intrinsic emission spectra of emitting layers). The overall far-field radiation characteristics into the high-index filler region or into air can then be obtained by averaging the contributions over these distributions**.**

The stack of OLED active layers are assumed to have the general structure of thick Ag bottom reflective electrode (150 nm)/hole-transport layer-HTL (y nm)/emitting layer-EML (10 nm)/electron-transport layer-ETL (x nm)/(semi-)transparent top electrode (either 20 nm Ag or 100 nm ITO). Actual optical constants [n(λ), k(λ)] of organic layers, Ag, and ITO were used in optical simulation [see **Figure**

S2(b) in **supplementary information**]. For simplicity of simulation, the optical properties (refractive index n) of the typical host material CBP (n~1.81 at 520 nm) are assumed for all the HTL, EML, ETL, and filler materials.

Step 2:

The optical reflectance, $R_s(\lambda,\theta_{AOI})$ and $R_p(\lambda, \theta_{AOI})$, and optical transmittance, $T_s(\lambda,\theta_{AOI})$, $T_p(\lambda,\theta_{AOI})$, seen from the high-index filler as a function of the polarization (s, p polarization), wavelength λ, and angle of incidence (θ_{AOI}) for each surface (bottom surface, bank slope surface) of the concave structure, that are needed for ray-tracing simulation in **Step 3**, are also calculated with the analytical electromagnetic wave theory.

For the bottom surface, the layer structures of Ag bottom electrode (150 nm)/CBP (of corresponding thickness)/(semi-)transparent top electrode (either 20 nm Ag or 100 nm ITO) for **devices 1-3** were used to calculate the optical reflection and transmission seen from the high-index filler. For simplicity, the additional dielectric layer that may be needed to define the actual emission aperture in implementation of the R-bank structure is omitted in most of simulation and discussion, since simulation shows very similar results with or without this additional dielectric layer (see supplementary **Figure S11-S12** and **Table S2**). Thus for calculating reflection and transmission of the R-bank slope surface, the layer structures similar to the bottom surface, except for all layer thicknesses being multiplied by $\cos\theta_B$ (θ_B is the bank angle) for taking into account the effect of oblique-angle deposition of material layers onto the bank slope [i.e., Ag bottom electrode ($150\times\cos\theta_B$ nm)/CBP (of corresponding thickness$\times\cos\theta_B$)/(semi-)transparent top electrode (either $20\times\cos\theta_B$ nm Ag or $100\times\cos\theta_B$ nm ITO) for **devices 1-3**], were used to calculate the optical reflection and transmission of the bank slope surface seen from the high-index filler. Meanwhile, the layer structures of semi-infinite D-bank/CBP (of corresponding thickness$\times\cos\theta_B$)/(semi-)transparent top electrode (either $20\times\cos\theta_B$ nm Ag or $100\times\cos\theta_B$ nm ITO) for **devices 1-3** were used to calculate the optical reflection and transmission of the D-bank slope surface seen from high-index filler.

Step 3:

With setting up ray sources (with results of **step 1**), surface optical properties (with results of **step 2**) and the geometric structure of the concave structure, the 3-dimensional (3D) polarization Monte Carlo ray-tracing simulation with the LightTools^{TM} software (Synopsys, Inc) is then conducted to calculate extraction of the light from the high-index filler region, taking into consideration the distribution of ray sources as a function of the wavelength λ, polarization (s, p polarization), and initial internal angle θ_{int}. Eventually the overall light extraction efficiency from the high-index filler to

air (i.e., η_{air}) and the overall out-coupled far-field emission intensity in air [$I(\lambda, \theta)$] as a function the wavelength λ and the external viewing angle θ.

The polarization ray-tracing calculus is adopted in the ray-tracing simulation so that the evolution of the polarization state throughout the whole optical process can be traced and polarization-dependent optical effects (such as intensity distributions of light sources, optical reflectance and transmittance of surfaces, Fresnel loss etc.) can be well taken into account for more accurate simulation and results. In the polarization ray-tracing calculus, the global coordinate system (with specified x, y, z axes as shown in **Figure S3(a)**) are used to keep track of evolution of various vector directions (e.g., the ray propagation vector **k**, the electric field (polarization) vector **E**) throughout the whole optical process. Meanwhile the local coordinate system for each optical surface/interface, as illustrated in **Figure S3(b)**, are used to unambiguously partition the polarization states (s or p) of the light ray incident onto that surface/interface and to treat the polarization-dependent processes (e.g., R_s, R_p, T_s, and T_p) at that surface/interface separately. The local coordinate system consists of three orthogonal vectors: the propagation vector (**k**), the vector perpendicular to the incident plane (s polarization direction), and the vector parallel to the incident plane (p polarization direction). As shown in **Figure S3(b)**, considering a linearly polarized ray with the propagation vector **k** and the electric field vector **E** incident on a surface, characteristics of reflected and transmitted rays can be determined by partitioning **E** into the s component $\mathbf{E}_{\mathbf{s,\,local}}$ and the p component $\mathbf{E}_{\mathbf{p,\,local}}$ relative to the local coordinate system, treating each component with the polarization-dependent reflectance ($\mathbf{R_s}$, $\mathbf{R_p}$) and transmittance ($\mathbf{T_s}$, $\mathbf{T_p}$) of that surface (calculated in **step 2**), and then combining them to get the electric field vector **E'** (polarization) of the reflected or transmitted ray (with the propagation vector **k'**) in the global coordinate system that would propagate toward the next surface/interface and undergo another optical process. By repeatedly tracing rays with vector operations alternately in the local and global coordinates till they are out-coupled to the air or lost at some point, the polarization-dependent optical characteristics of the optical system are assessed.

The ray source used for the 3D polarization ray-tracing simulation in **step 3** is set up based on calculated radiation intensity distributions $I_s(\lambda, \theta_{int})$, $I_p(\lambda, \theta_{int})$ coupled to the filler region, that are calculated in **step 1** and contain the complete wavelength, angular and polarization dependence. In consideration of the singularity issue in the polarization ray-tracing calculus[9,10], the ray source in this study is built by using a user-defined polarizer to ensure that the rays pass through it will have the same polarization characteristics calculated from the electromagnetic wave method in **step 1**. Although the Monde Carlo ray-tracing method works efficiently with increasing the number of rays, consideration of the computation loading limits the initial ray quantity to 200 million in this study. The intensity distributions and the efficiency in air are collected by an enclosed sphere with infinite

radius. To avoid collection of the leakage modes and emission towards lateral sides of the structure and to ensure only collection of emission toward the forward direction, the lateral side surfaces and the bottom surfaces of the structure are set to be absorptive in our simulation model. The angular spectra are collected by apertures of 1° full cone angle at corresponding angles (e.g., 0°, 30°, 60° etc.). Through the comprehensive 3D polarization ray-tracing simulation with complete consideration of angular, spectral, and polarization dependence of ray sources, the light extraction efficiency from the high-index filler to air (i.e., η_{air}) and the far-field emission pattern in air $I(\lambda, \theta)$ can be obtained. The overall light extraction efficiency from the OLED active region to air (i.e., η_{ext}) can then be obtained by $\eta_{ext}=\eta_{filler} \times \eta_{air}$.

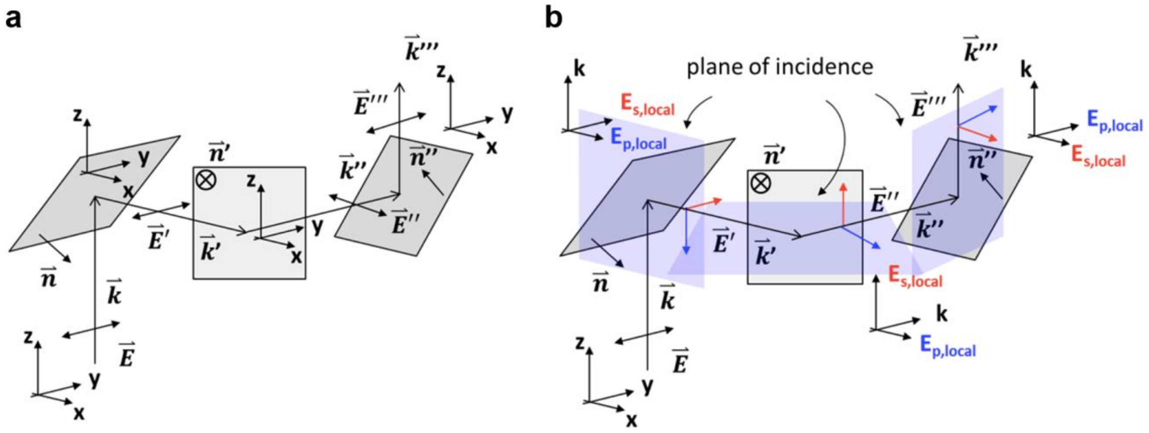

Figure S3. In polarization ray-tracing calculus, the vectors are represented in (a) global coordinates, and (b) local coordinates.

References

1. Neyts, K. A., Simulation of light emission from thin-film microcavities. *J. Opt. Soc. Am.* **15**, 962-971 (1998).
2. Lu, M.-H., Sturm, J. C. Optimization of external coupling and light emission in organic light-emitting devices: modeling and experiment. *J. Appl. Phys.* **91**, 595-604 (2002).
3. Wu, C.-C., Chen, C.-W., Lin, C.-L., Yang, C.-J. Advanced organic light-emitting devices for enhancing display performances. *J. Disp. Technol.* **1**, 248-266 (2005).
4. Lin, C. L., Cho, T. Y., Chang, C. H. & Wu, C. C. Enhancing light outcoupling of organic light-emitting devices by locating emitters around the second antinode of the reflective metal electrode. *Appl. Phys. Lett.* **88**, 81114 (2006).
5. Brütting, W., Frischeisen, J., Schmidt, T. D., Scholz, B. J., Mayr, C. Device efficiency of organic light-emitting diodes: Progress by improved light outcoupling. *Phys. Status Solidi A* **210**, 44–65 (2013).
6. Bahl, M., Zhou, G.-R., Heller, E., Cassarly, W., Jiang,M., Scarmozzino, R., Gregory, G. G. Optical simulations of organic light-emitting diodes through a combination of rigorous electromagnetic solvers and Monte Carlo ray-tracing methods. *Proc. SPIE* **9190**,919009 (2014).

7. Chang, K.-D., Li, C.-Y., Pan, J.-W., Cheng, K.-Y. A hybrid simulated method for analyzing the optical efficiency of a head-mounted display with a quasi-crystal OLED panel. *Opt. Express* **22**, A567-A576 (2014).
8. Chen, C.-Y., Chen, Y.-J., Lee, W.-K. Lu, C.-Y., Lin, H. Y., Wu, C.-C. Analyses of optical out-coupling of organic light- emitting devices having micromesh indium tin oxide and conducting polymer as composite transparent electrode. *Opt. Express* **24**, A810-A822 (2016).
9. Yun, G., Crabtree, K., Chipman, R. A. Properties of the polarization ray tracing matrix. *Proc. SPIE* **6682**, 66820Z (2007).
10. Yun, G., Crabtree, K., Chipman, R. A. Three-dimensional polarization ray-tracing calculus I: definition and diattenuation. *Appl. Optics* **50**, 2855-2865 (2011).

2. More simulation data

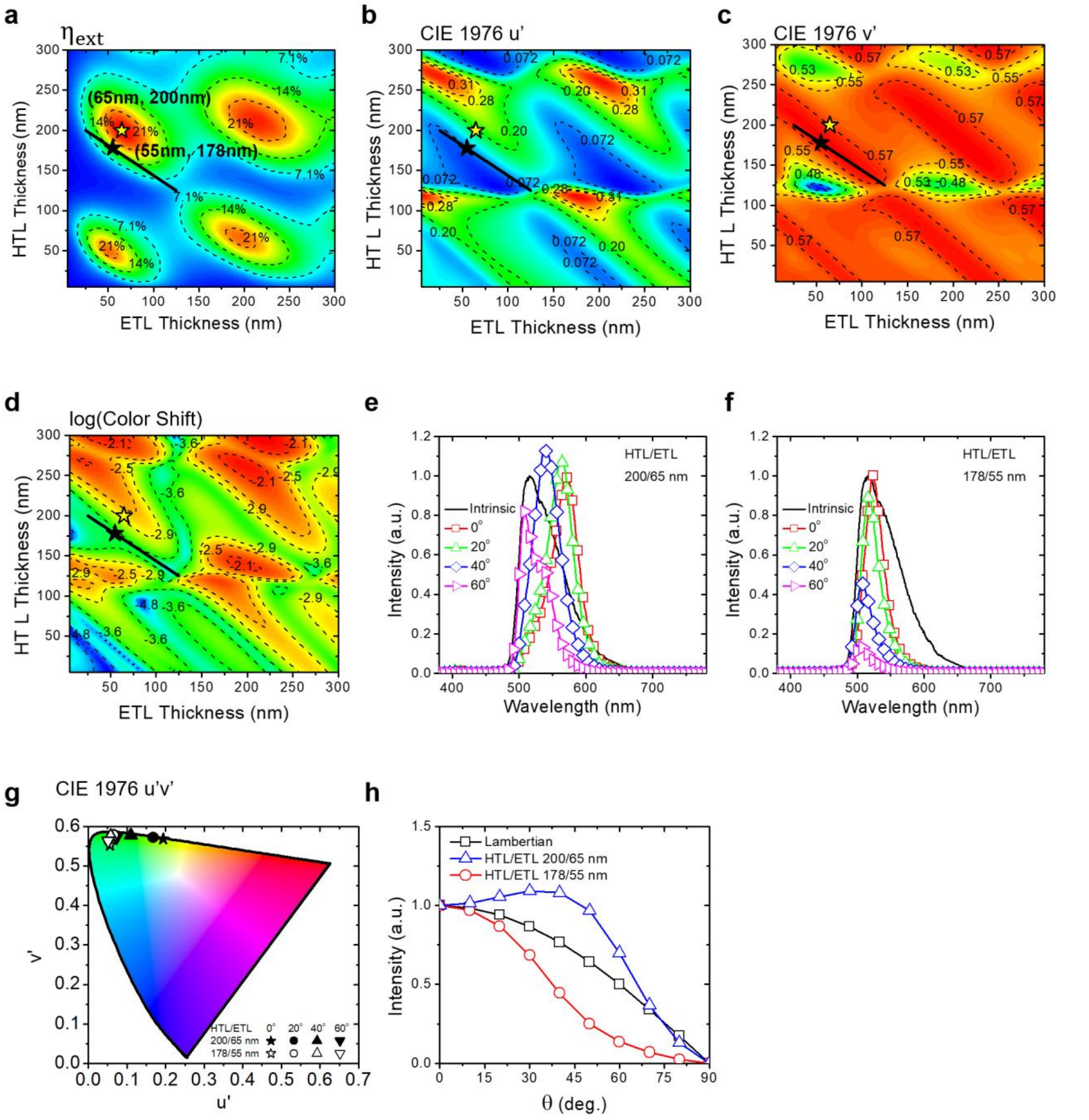

Figure S4. Emission characteristics of conventional top-emitting device with the thin Ag top electrode. The device has a general structure of: reflective Ag bottom electrode (150 nm)/HTL (y nm)/EML (10 nm)/ETL (x nm)/20-nm Ag/70-nm CBP capping, assuming isotropic green emitters in the EML. **(a)** Calculated light extraction efficiencies η_{ext} as a function of the HTL and ETL thicknesses. **(b)-(d)** Calculated CIE 1976 (u', v') color coordinates at the 0° viewing angle and the color shift as a function of the HTL and ETL thicknesses. The color shift is defined and calculated as [variance of u'(θ) + variance of v'(θ)], and measures variation of colors over viewing angles. The thick black lines in panels (a)-(d) represent the structures with more acceptable color performance (more acceptable CIE 1976 u', v' color coordinates at the 0° viewing angle and minimized color shift). The yellow star with (ETL, HTL)=(65 nm, 200 nm) in (a)-(d) represents the structure with highest η_{ext}, (~25-26%). The black star with (ETL, HTL)=(55 nm, 178 nm) on the thick black lines in (a)-(d) represents the structure

having a lower η_{ext} (~16%) compromised with more acceptable color performance. **(e)** Calculated emission spectra vs. external viewing angle for the device with highest η_{ext} (25-26%) in (a) (i.e., the yellow star with (ETL, HTL)=(65 nm, 200 nm)). **(f)** Calculated emission spectra vs. viewing angle for the device having a lower η_{ext} (~16%) compromised with more acceptable color performance (i.e., the black star with (ETL, HTL)=(55 nm, 178 nm) in (a)). **(g)** CIE 1976 (u', v') color coordinates at different viewing angles corresponding to spectra in (e) and (f). **(h)** Angular dependence of emission intensity for the two devices in (e) and (f), in comparison with the Lambertian pattern.

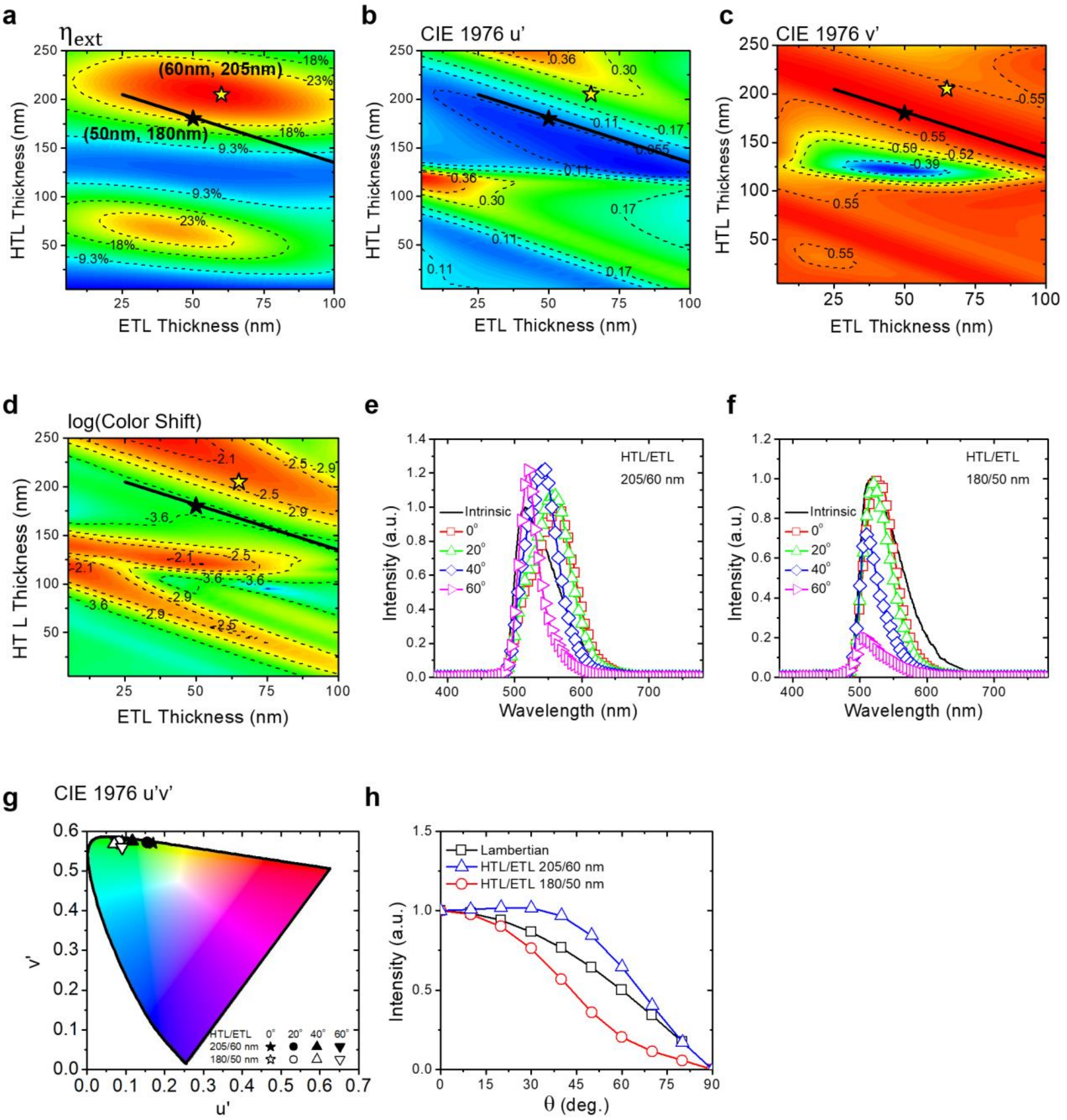

Figure S5. Emission characteristics of conventional top-emitting device with the ITO top electrode. The device has a general structure of: reflective Ag bottom electrode (150 nm)/HTL (y nm)/EML (10 nm)/ETL (x nm)/100-nm ITO, assuming isotropic green emitters in the EML. **(a)** Calculated light extraction efficiencies η_{ext} as a function of the HTL and ETL thicknesses. **(b)-(d)** Calculated CIE 1976 (u', v') color coordinates at the 0° viewing angle and the color shift as a function of the HTL and ETL thicknesses. The color shift is defined and calculated as [variance of u'(θ) + variance of v'(θ)], and measures variation of colors over viewing angles. The thick black lines in panels (a)-(d) represent the structures with more acceptable color performance (more acceptable CIE 1976 u', v' color coordinates at the 0° viewing angle and minimized color shift). The yellow star with (ETL, HTL)=(60 nm, 205 nm) in (a)-(d) represents the structure with highest η_{ext}, (~30.3%). The black star with (ETL, HTL)=(50 nm, 180 nm) on the thick black lines in (a)-(d) represents the structure having a lower η_{ext} (~23.8%) compromised with more acceptable color performance. **(e)** Calculated emission spectra vs. viewing angle for the device with highest η_{ext} (30.3%) in (a) (i.e., the yellow star with (ETL, HTL)=(60 nm, 205 nm)). **(f)** Calculated emission spectra vs. viewing angle for the device having a lower η_{ext}

(~23.8%) compromised with acceptable color performance (i.e., the black star with (ETL, HTL)=(50 nm, 180 nm) in (a)). **(g)** CIE 1976 (u', v') color coordinates at different viewing angles corresponding to spectra in (e) and (f). **(h)** Angular dependence of emission intensity for the two devices in (e) and (f), in comparison with the Lambertian pattern.

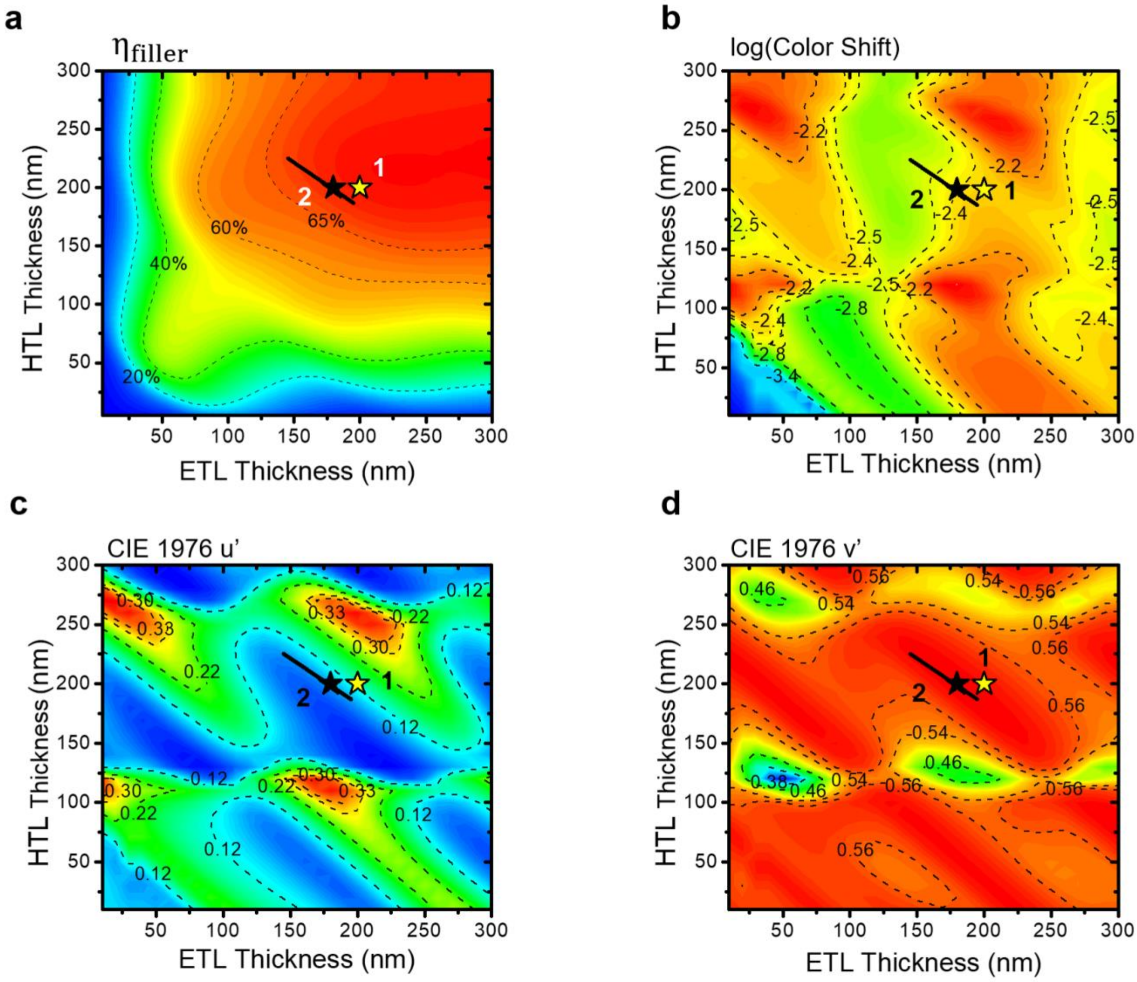

Figure S6. Characteristics of emission coupled into the filler for the device with the thin Ag top electrode. The device has a general structure of: reflective Ag bottom electrode (150 nm)/HTL (y nm)/EML (10 nm)/ETL (x nm)/20-nm Ag/semi-infinite CBP, assuming isotropic green emitters in the EML. **(a)** Calculated coupling efficiency into the filler η_{filler} as a function of the HTL and ETL thicknesses. **(b)-(d)** Calculated CIE 1976 (u', v') color coordinates at the internal angle θ_{int}=0° and the color shift as a function of the HTL and ETL thicknesses. The color shift is defined and calculated as [variance of u'(θ_{int}) + variance of v'(θ_{int})], and measures variation of colors over internal angles θ_{int}. The thick black lines in panels (a)-(d) represent the structures with more acceptable color performance (acceptable CIE 1976 (u', v') color coordinates at the θ_{int}=0° internal angle and minimized color shift) in the filler. The yellow star with (ETL, HTL)=(200 nm, 200 nm) in (a)-(d) represents the structure with highest η_{filler}, (~69.8%). The black star with (ETL, HTL)=(180 nm, 200 nm) on the thick black lines in (a)-(d) represents the structure having a lower η_{filler} (~68.6%) compromised with more acceptable color performance in the filler.

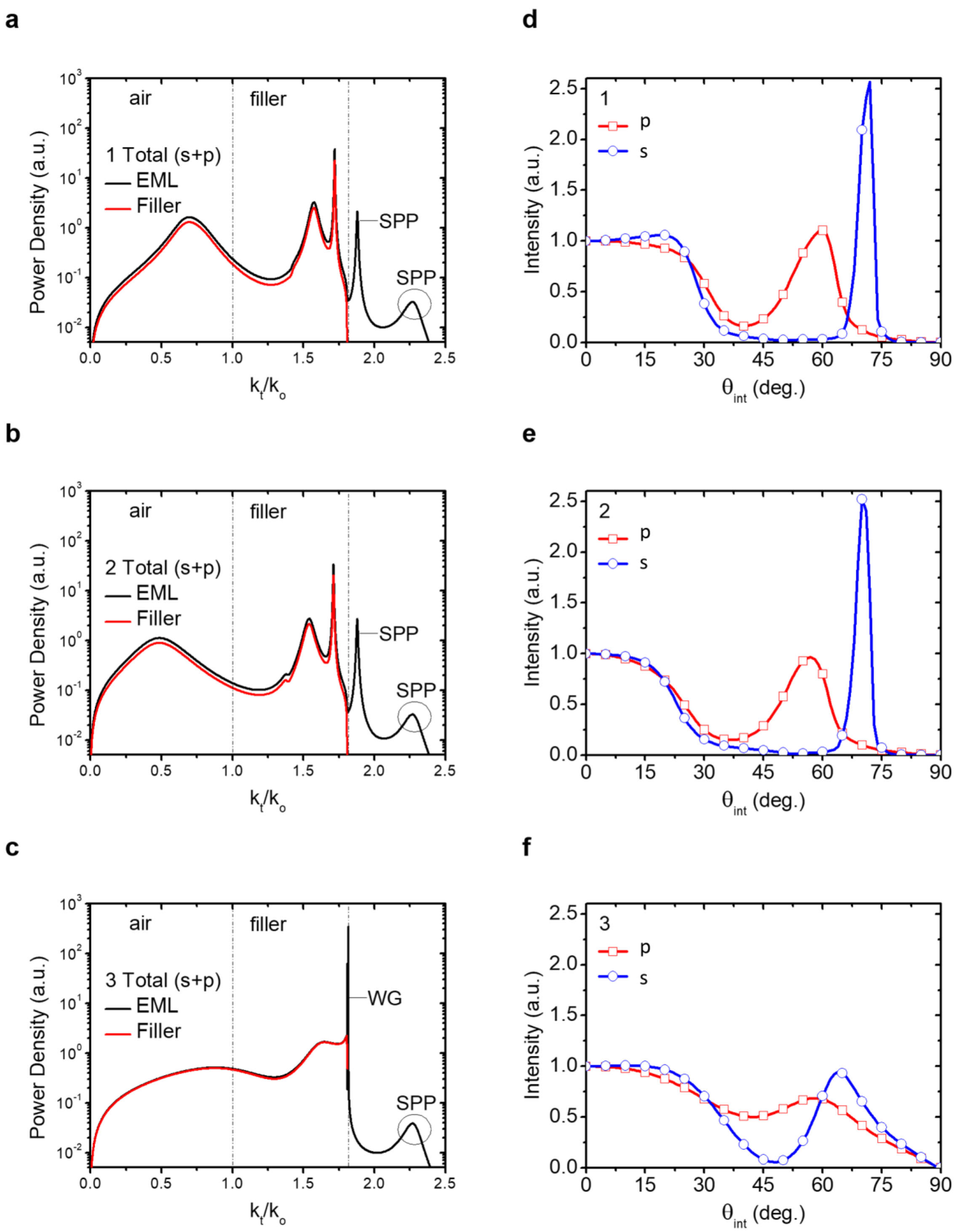

Figure S7. (a)-(c) Optical modal distributions of radiation from devices 1-3 in the emitting layer (EML) and into the filler as a function of k_t/k_o (k_t is the in-plane wavevector in the device and k_o is the free-space wavevector) with assuming a semi-infinite filler. In **devices 1/2**, the reduction of modal intensities from EML to filler for the k_t/k_o range of 0 - ~1.8 are associated with the absorption loss by electrodes and losses induced by Fresnel reflection at organic-electrode interfaces when light propagating from the OLED electrode-organic-electrode region toward the filler region. **(d)-(f)**

Polarization-resolved spectrally integrated emission patterns (p- and s-polarization components) for **device 1**-**3** emission coupled into the high-index filler, respectively.

Table S1. Summary of optical mode distribution ratios for devices 1-3 in the 3D pixel structure.

Device	SPP[a)] (%)	WG+loss[b)] (%)	Air[c)] (%)	Filler[d)] (%)	
				Filler-to-air[e)] (%)	Absorption loss[f)] (%)
1	4.4	25.8	26.4	43.4	
				22.8	20.6
2	5.7	25.7	21.9	46.7	
				25.2	21.5
3	3.9	8.3	24.7	63.1	
				47.9	15.2

[a] surface plasmon modes (SPP). [b] waveguided modes and losses (e.g. absorption by electrodes, losses induced by Fresnel reflection at organic-electrode interfaces) associated with the OLED electrode-organic-electrode structure (WG+loss). [c] air (radiation) modes that can be out-coupled to air directly. [d] filler modes that are coupled to the filler but would otherwise be confined and waveguided in the filler if there is no further extraction scheme. [e] the portion of filler modes that can be further extracted to air by the 3D R-bank structure. [f] the portion of filler modes that is subjected to absorption loss during propagation and reflection in the structure.

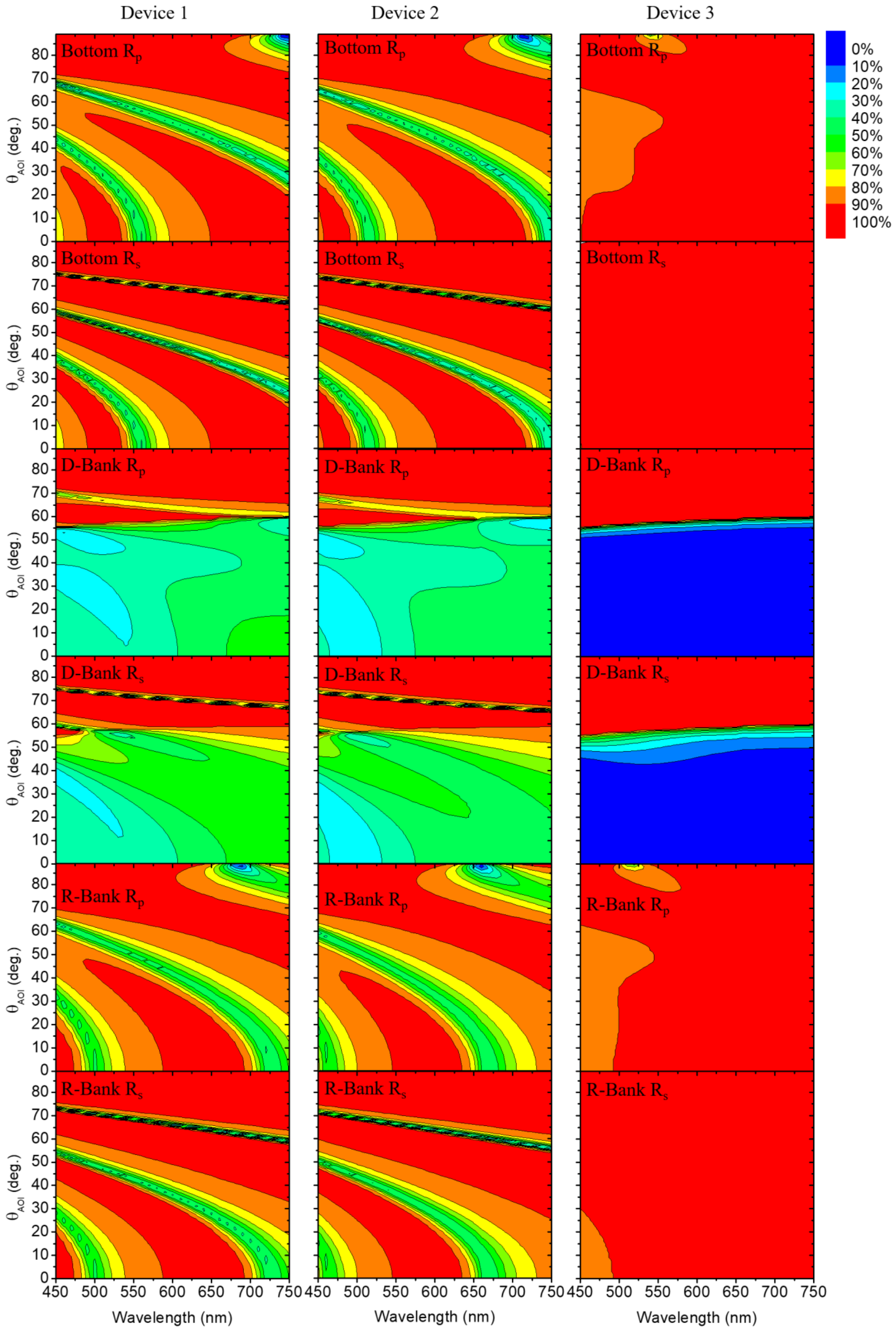

Figure S8. Optical reflection properties of surfaces in various bank+filler structures. Calculated $R_s(\lambda, \theta_{AOI})$ and $R_p(\lambda, \theta_{AOI})$ for each surface (bottom surface and bank slope surface) of the concave structure, for **device 1**, **2**, **3** in either the D-bank+filler structure or the R-bank+filler structure having

θ_B.=30° For the bottom surface, the layer structures of Ag bottom electrode (150 nm)/CBP (of corresponding thickness)/(semi-)transparent top electrode (either 20 nm Ag or 100 nm ITO) for **devices 1-3** were used to calculate R_s and R_p. For calculating reflection of the R-bank slope surface, the layer structures similar to the bottom surface, except for all layer thicknesses being multiplied by $\cos\theta_B$ for taking into account the effect of oblique-angle deposition (i.e., Ag bottom electrode ($150\times\cos\theta_B$ nm)/CBP (of corresponding thickness$\times\cos\theta_B$)/(semi-)transparent top electrode (either $20\times\cos\theta_B$ nm Ag or $100\times\cos\theta_B$ nm ITO) for **devices 1-3**), were used. Meanwhile, the layer structures of semi-infinite D-bank/CBP (of corresponding thickness$\times\cos\theta_B$)/(semi-)transparent top electrode (either $20\times\cos\theta_B$ nm Ag or $100\times\cos\theta_B$ nm ITO) were used to calculate the reflection of the D-bank slope surface seen from high-index filler for **devices 1-3**.

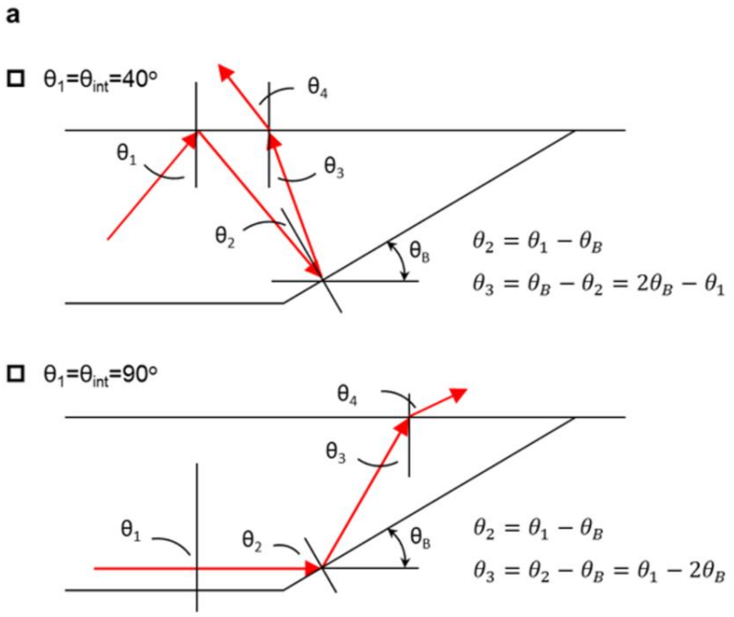

Ray-tracing Table (θ_B=30°)

$\theta_1=\theta_{int}$	θ_2	θ_3	$\theta_4=\theta$
40°	10°	20°	38.3°
50°	20°	10°	18.3°
60°	30°	0°	0°
70°	40°	10°	18.3°
80°	50°	20°	38.3°
90°	60°	30°	64.8°

$\theta_1=\theta_{int}$=initial internal angle in filler
$\theta_4=\theta$=external viewing angle

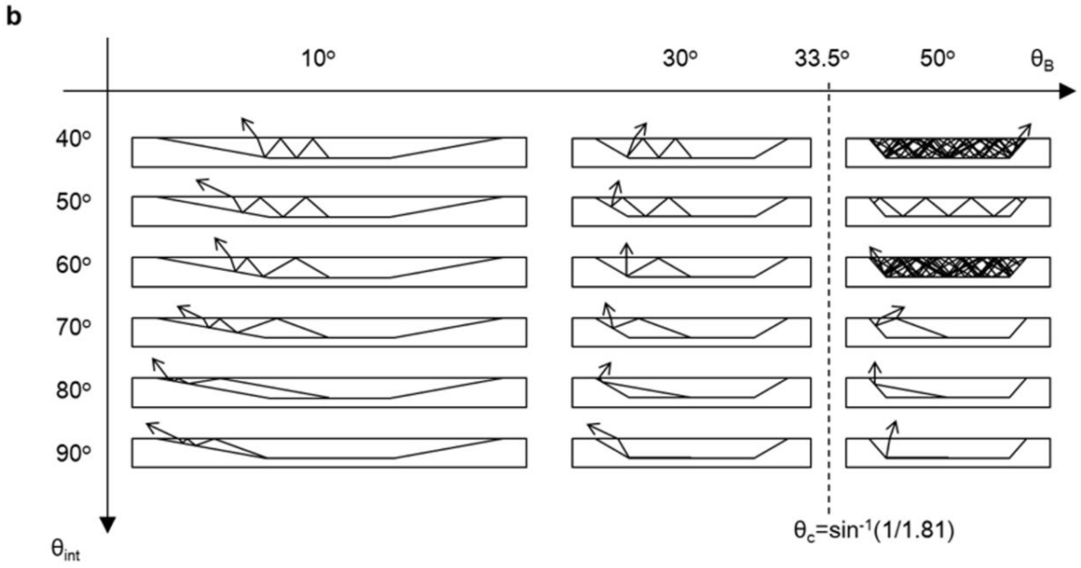

Figure S9. Illustration of re-direction and bouncing reflection of light rays in the bank+filler structure. (a) Illustrations of how light rays with any initial θ_{int} >θ_c of the filler-air interface are re-directed into the θ_c escape cone for out-coupling in the case with the bank angle θ_B =30°. **(b)** Traces of bouncing reflection of various light rays with different initial θ_{int} >θ_c in the bank+filler structures with different θ_B.

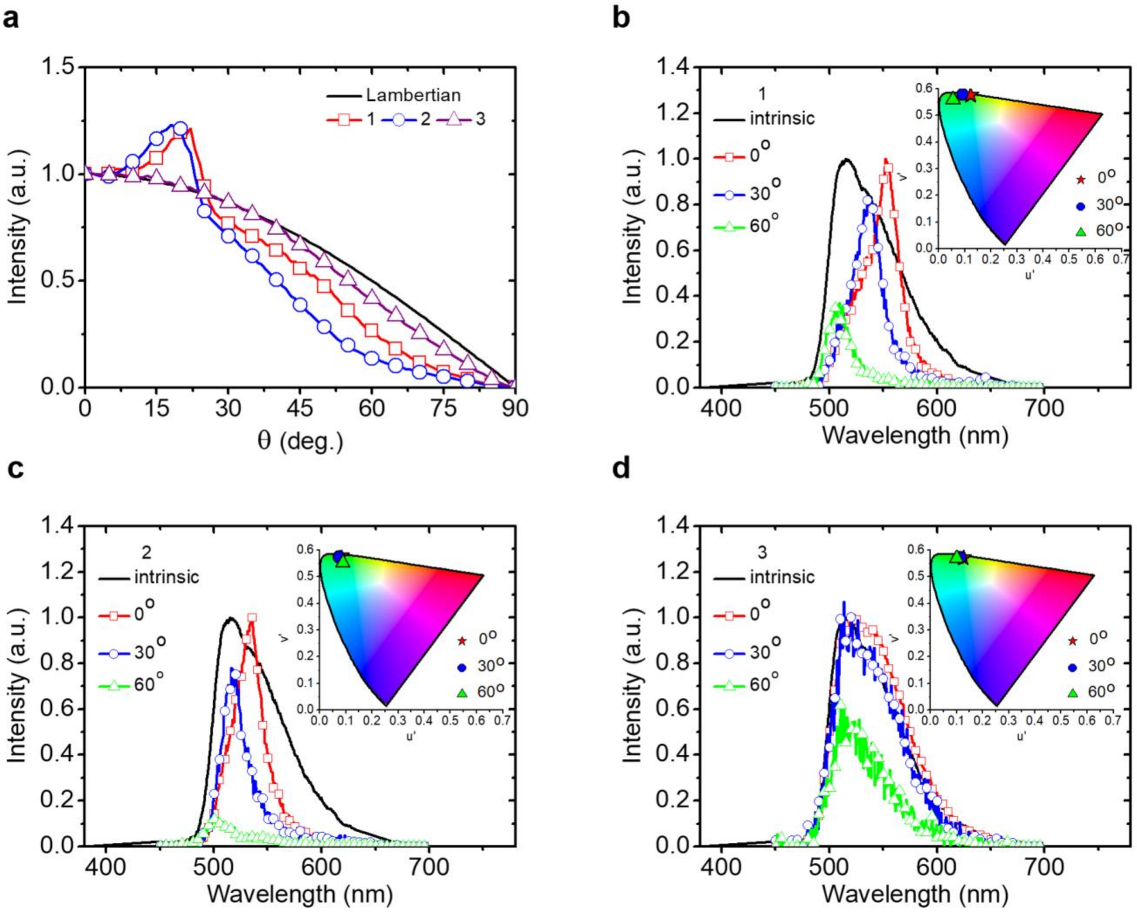

Figure S10. Out-coupled pixel OLED emission for D-bank+filler structures. H=2 μm, θ_B =30°, W_1=13 μm, d=1 μm, $2W_2$~7 μm, $2W_3$~5 μm, p=25 μm are assumed. **(a)** Viewing-angle dependent (spectrally integrated) out-coupled emission intensity for devices **1**, **2**, **3** in the D-bank+filler structure. The Lambertian pattern is also shown for comparison. **(b)-(d)** Viewing-angle dependent out-coupled emission spectra for devices **1**, **2**, **3** in the D-bank+filler structure. The intrinsic emission spectrum of the emitter (isotropic green emitter) is also shown for comparison.

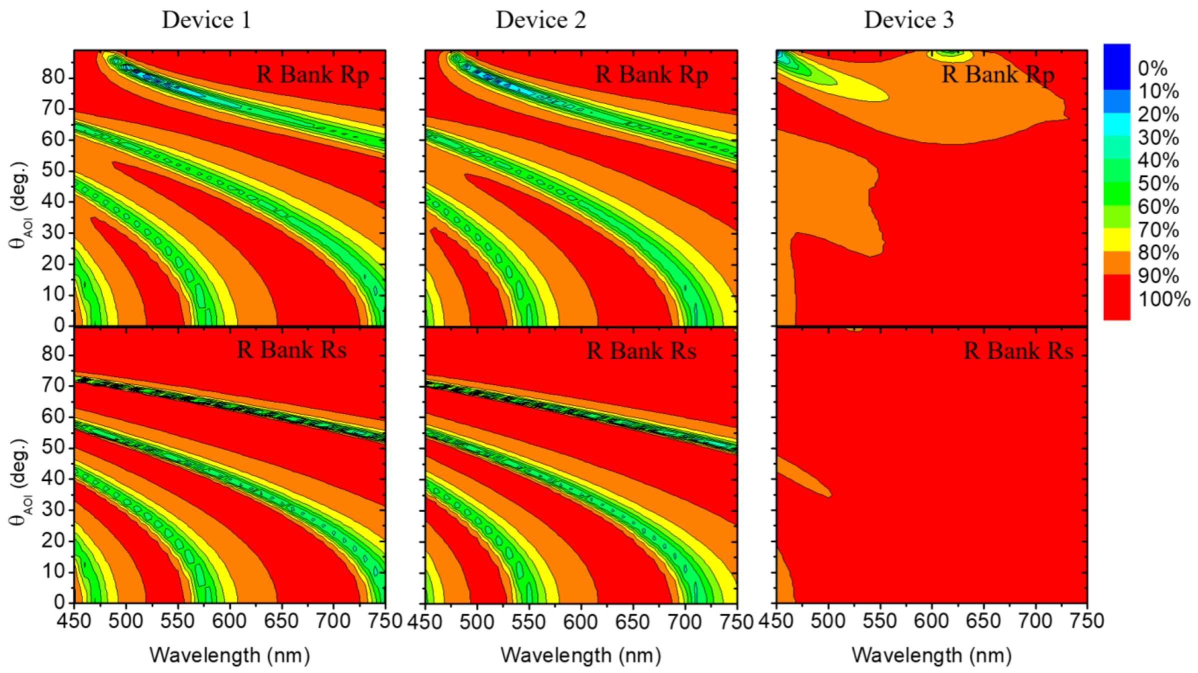

Figure S11. Optical reflection properties of surfaces of R-bank+filler structures with additional dielectric layer over the bank slope reflector. Calculated $R_s(\lambda, \theta_{AOI})$ and $R_p(\lambda, \theta_{AOI})$ for the R-bank slope surface for **device 1**, **2**, **3** in the R-bank+filler structure having θ_B.=30°, with the additional dielectric layer (assuming 200-nm SiNx) coated over the bank slope portion of the bottom Ag electrode for insulation and for defining the emission aperture at the bottom surface of the concave structure. For calculating reflection of the R-bank slope surface, the layer structures of: Ag bottom electrode (150×cosθ_B nm)/200-nm SiNx/CBP (of corresponding thickness×cosθ_B)/(semi-)transparent top electrode (either 20×cosθ_B nm Ag or 100×cosθ_B nm ITO) for **devices 1-3**, were used. R_s and R_p of the bottom surface and the D-bank surface for **devices 1-3**, are same those in **Figure S8** and are not shown here.

Table S2. Comparison of light coupling/extraction efficiencies for the R-bank structures with or without the additional dielectric layer (200-nm SiNx) coated over the bank slope for insulation and for defining the emission aperture at the bottom surface.

Device	Additional Dielectric Layer	η_{filler} (%)	η_{air} (%)	η_{ext} (%)
1	w/o	69.8	70.6	49.2
	w/		69.6	48.6
2	w/o	68.6	68.7	47.1
	w/		67.3	46.1
3	w/o	87.8	82.6	72.6
	w/		81.4	71.4
R Bank, Color=Green, HR=67%, $\theta_B = 30^o$, H= 2 μm, W_1=13 μm				

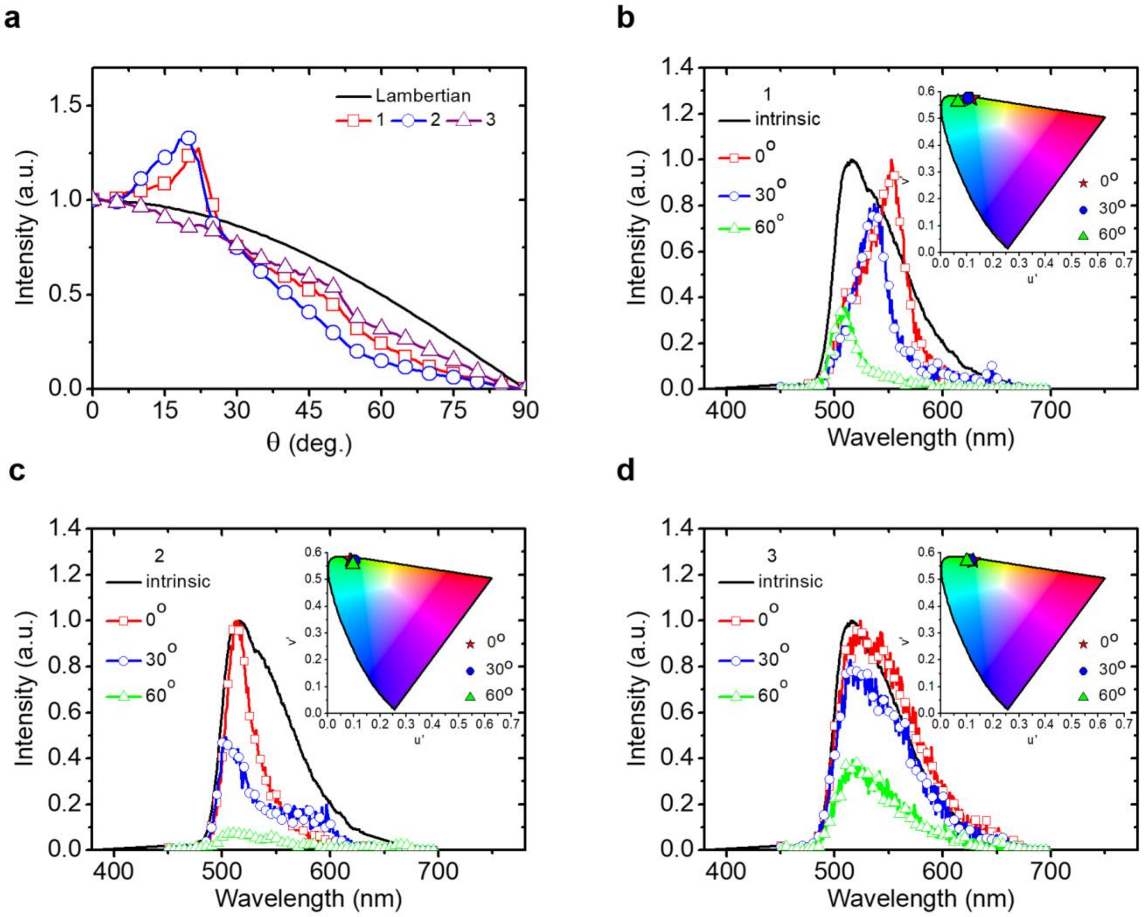

Figure S12. Out-coupled pixel OLED emission for R-bank+filler structures with additional dielectric layer over the bank slope reflector. H=2 μm, θ_B =30°, W_1=13 μm, d=1 μm, $2W_2$~7 μm, $2W_3$~5 μm, p=25 μm are assumed. **(a)** Out-coupled viewing-angle dependent (spectrally integrated) out-coupled emission intensity for devices **1**, **2**, **3** in the R-bank+filler structure. The Lambertian pattern is also shown for comparison. **(b)-(d)** Viewing-angle dependent out-coupled emission spectra for devices **1**, **2**, **3** in the R-bank+filler structure. The intrinsic emission spectrum of the emitter (assume isotropic green emitter) is also shown for comparison.

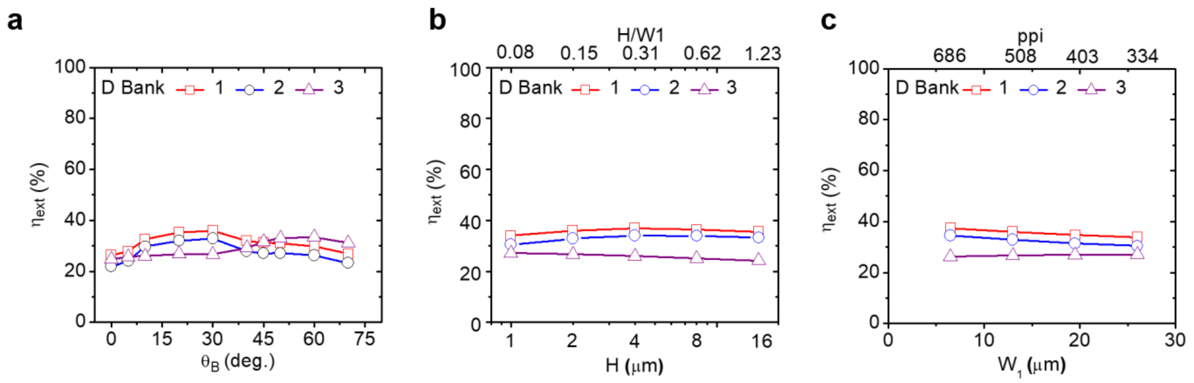

Figure S13. Influences of structures on out-coupling efficiency of the D-bank+filler structure. (a) Calculated η_{ext} as a function of the bank angle θ_B for **devices 1-3** in the D-bank+filler structure with H=2 μm, W_1=13 μm, d=1 μm, $2W_3$=5 μm. **(b)** Calculated η_{ext} as a function of the bank height H for **devices 1-3** in the D-bank+filler structure with θ_B =30°, W_1=13 μm, d=1 μm, $2W_3$=5 μm. **(c)** Calculated η_{ext} as a function of the bottom width W_1 for **devices 1-3** in the D-bank+filler structure. H=2 μm, θ_B =30°, d=1 μm, $2W_2$=7 μm, $2W_3$=5 μm.

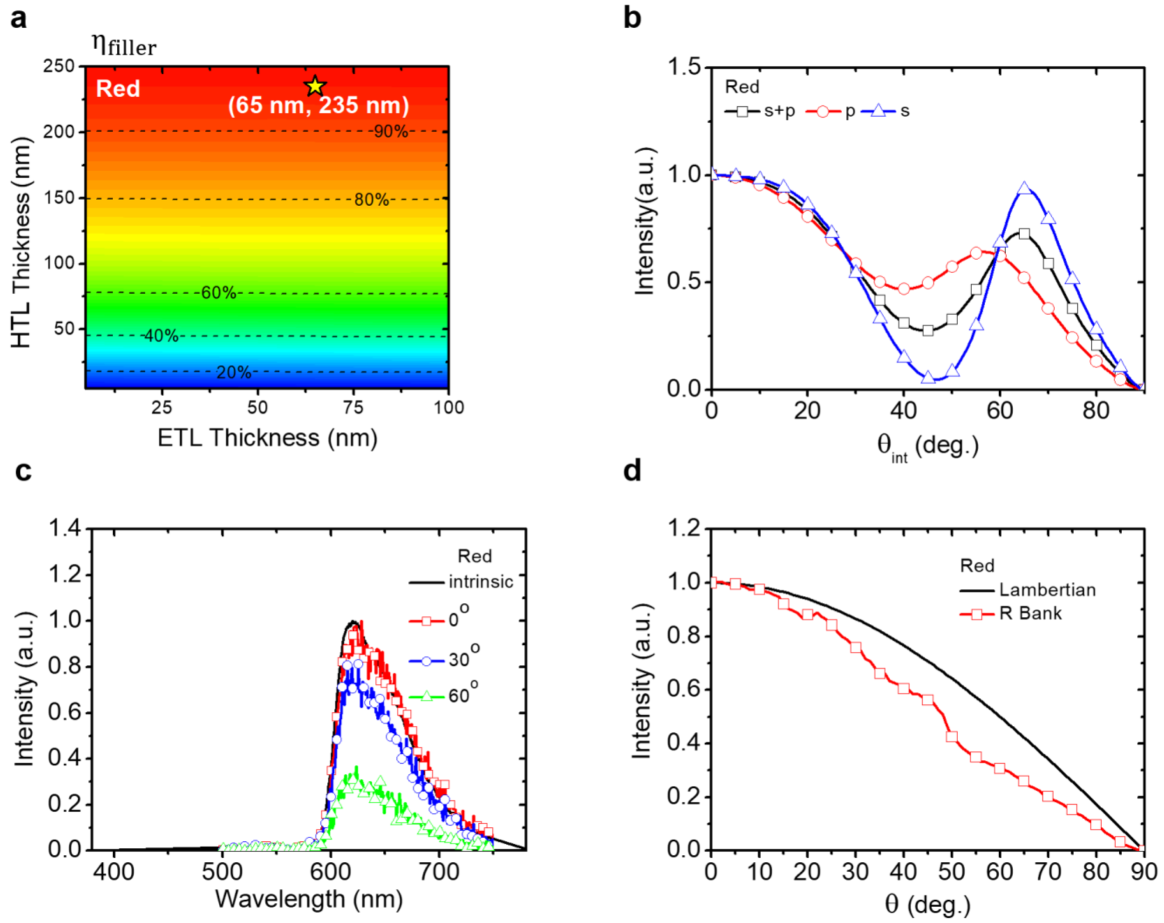

Figure S14. Characteristics of red-emitting device with the ITO top electrode (red device 3) in the R-bank+filler structure. H=2 μm, θ_B =30°, W_1=13 μm, d=1 μm, $2W_2$~7 μm, $2W_3$~5 μm, p=25 μm are assumed. **(a)** Calculated η_{filler}, the coupling efficiency of the radiation generated in the OLED active region to the high-index filler, as a function of the HTL and ETL thicknesses for the ITO device: Ag(150 nm)/HTL(y nm)/EML(10 nm)/ETL(x nm)/ITO (100 nm)/CBP (semi-infinite), assuming isotropic red emitters in the EML. The yellow star with (ETL, HTL)=(65 nm, 235 nm) in (a) represents the structure with highest η_{filler} (~93.7%). **(b)** Spectrally integrated radiation patterns coupled into the filler region for the the device with highest η_{filler}. **(c)** Viewing-angle dependent out-coupled emission spectra for the highest η_{filler} device in the R-bank+filler structure. The hypothetical intrinsic emission spectrum of the red emitter is also shown for comparison. **(d)** Out-coupled viewing-angle dependent (spectrally integrated) emission intensity for the highest η_{filler} device in the R-bank+filler structure. The Lambertian pattern is also shown for comparison.

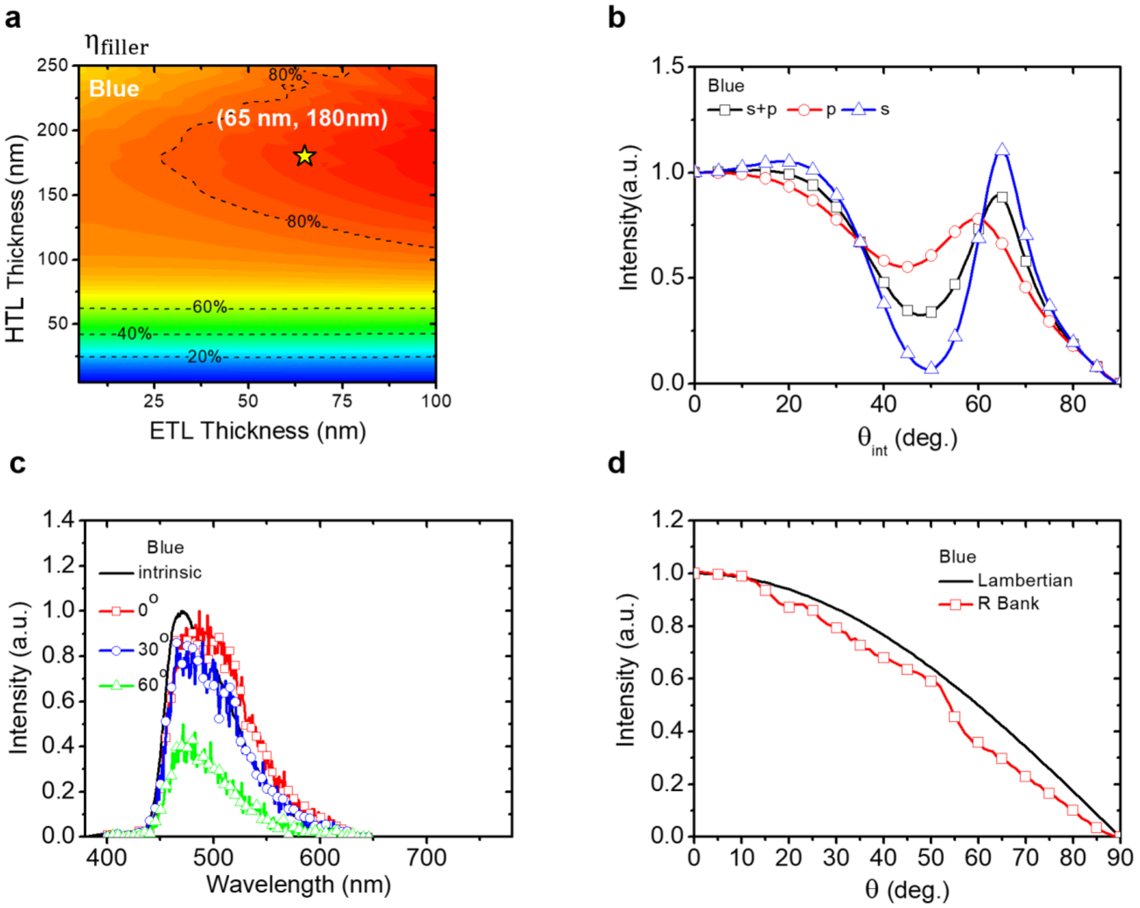

Figure S15. Characteristics of blue-emitting device with the ITO top electrode (blue device 3) in the R-bank+filler structure. H=2 μm, θ_B =30°, W_1=13 μm, d=1 μm, $2W_2$~7 μm, $2W_3$~5 μm, p=25 μm are assumed. **(a)** Calculated η_{filler}, the coupling efficiency of the radiation generated in the OLED active region to the high-index filler, as a function of the HTL and ETL thicknesses for the ITO device: Ag(150 nm)/HTL(y nm)/EML(10 nm)/ETL(x nm)/ITO (100 nm)/CBP (semi-infinite), assuming isotropic blue emitters in the EML. The yellow star with (ETL, HTL)=(65 nm, 180 nm) in (a) represents the structure with highest η_{filler} (~83.5%). **(b)** Spectrally integrated radiation patterns coupled into the filler region for the thr device with highest η_{filler}. **(c)** Viewing-angle dependent out-coupled emission spectra for the highest η_{filler} device in the R-bank+filler structure. The hypothetical intrinsic emission spectrum of the blue emitter is also shown for comparison. **(d)** Out-coupled viewing-angle dependent (spectrally integrated) emission intensity for the highest η_{filler} device in the R-bank+filler structure. The Lambertian pattern is also shown for comparison.

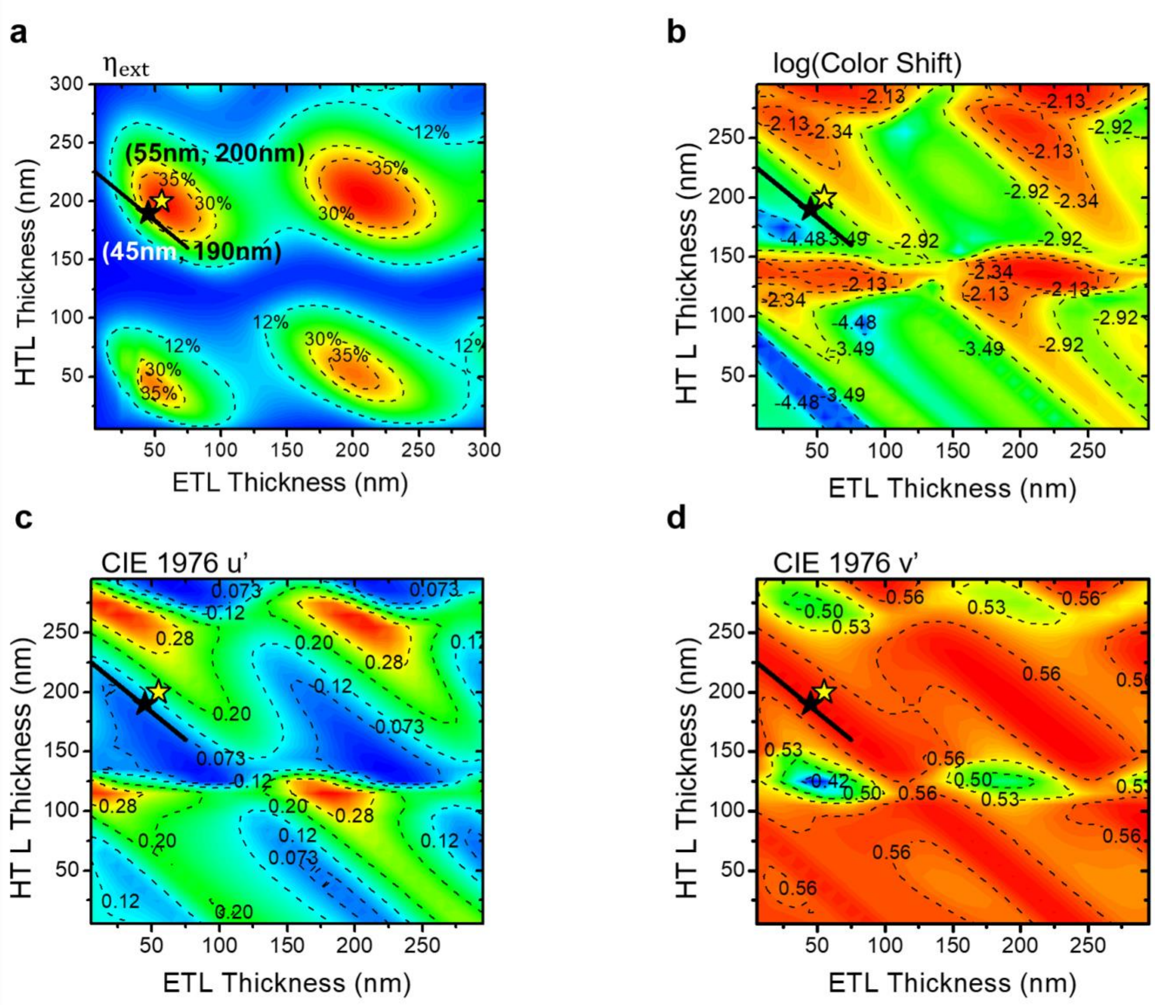

Figure S16. Emission characteristics of conventional top-emitting device with the thin Ag top electrode and 100% horizontal dipole emitters. The device has a general structure of: reflective Ag bottom electrode (150 nm)/HTL (y nm)/EML (10 nm)/ETL (x nm)/20-nm Ag/70-nm CBP capping, assuming green emitters with a 100% horizontal dipole ratio in the EML. **(a)** Calculated light extraction efficiencies η_{ext} as a function of the HTL and ETL thicknesses. **(b)-(d)** Calculated CIE 1976 (u', v') color coordinates at the 0° viewing angle and the color shift as a function of the HTL and ETL thicknesses. The color shift is defined and calculated as [variance of u'(θ) + variance of v'(θ)], and measures variation of colors over viewing angles. The thick black lines in panels (a)-(d) represent the structures with more acceptable color performance (more acceptable CIE 1976 u', v' color coordinates at the 0° viewing angle and minimized color shift). The yellow star with (ETL, HTL)=(55 nm, 200 nm) in (a)-(d) represents the structure with highest η_{ext}, (41.3%). The black star with (ETL, HTL)=(45 nm, 190 nm) on the thick black lines in (a)-(d) represents the structure having a lower η_{ext} (~34.1%) compromised with more acceptable color performance.

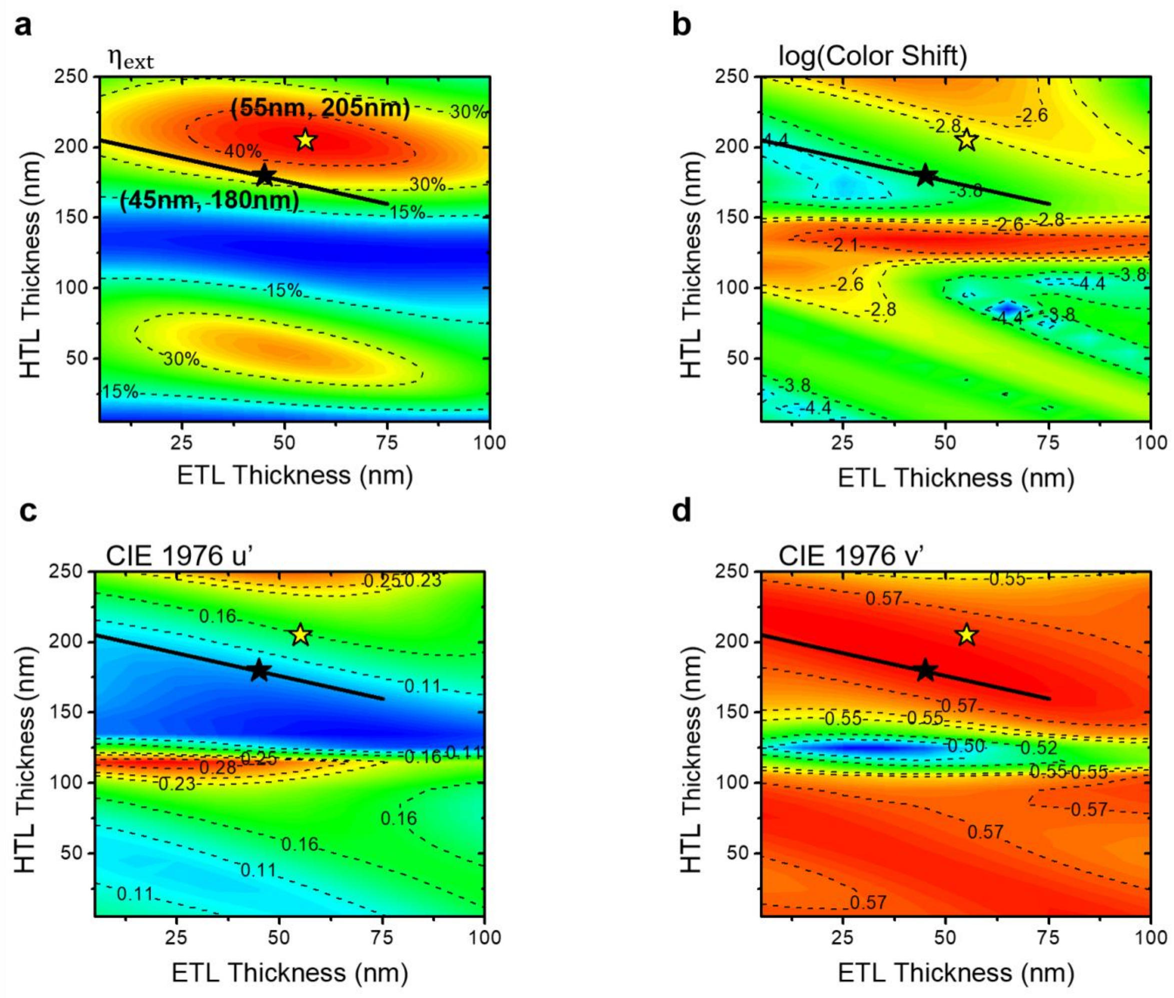

Figure S17. Emission characteristics of conventional top-emitting device with the ITO top electrode and 100% horizontal dipole emitters. The device has a general structure of: reflective Ag bottom electrode (150 nm)/HTL (y nm)/EML (10 nm)/ETL (x nm)/100-nm ITO, assuming green emitters with a 100% horizontal dipole ratio in the EML. **(a)** Calculated light extraction efficiencies η_{ext} as a function of the HTL and ETL thicknesses. **(b)-(d)** Calculated CIE 1976 (u', v') color coordinates at the 0° viewing angle and the color shift as a function of the HTL and ETL thicknesses. The color shift is defined and calculated as [variance of u'(θ) + variance of v'(θ)], and measures variation of colors over viewing angles. The thick black lines in panels (a)-(d) represent the structures with more acceptable color performance (more acceptable CIE 1976 u', v' color coordinates at the 0° viewing angle and minimized color shift). The **yellow** star with (ETL, HTL)=(**55 nm, 205 nm)** in (a)-(d) represents the structure with highest η_{ext}, (~**43.3**%). The **black** star with (ETL, HTL)=(**45 nm, 180** nm) on the thick black lines in (a)-(d) represents the structure having a lower η_{ext} (~**34.5**%) compromised with more acceptable color performance.

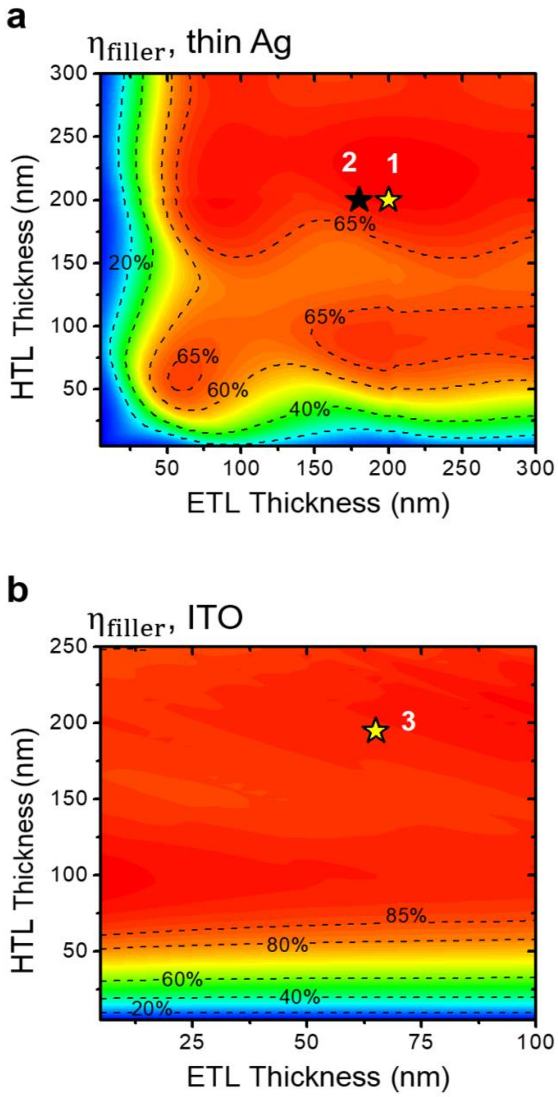

Figure S18. Calculated η_{filler} for green-emitting devices with 100% horizontal dipole emitters. (a) Calculated η_{filler}, the coupling efficiency of the radiation generated in the OLED active region to the high-index filler, as a function of the HTL and ETL thicknesses for the device with thin Ag top electrode: Ag(150 nm)/HTL(y nm)/EML(10 nm)/ETL(x nm)/Ag (20 nm)/CBP (semi-infinite), assuming 100% horizontal dipole emitters (green emitting) in the EML. The yellow star with (ETL, HTL)=(200 nm, 200 nm) in (a) represents the structure with highest η_{filler} (~70%). (b) Calculated η_{filler}, the coupling efficiency of the radiation generated in the OLED active region to the high-index filler, as a function of the HTL and ETL thicknesses for the device with ITO top electrode: Ag(150 nm)/HTL(y nm)/EML(10 nm)/ETL(x nm)/ITO (100 nm)/CBP (semi-infinite), assuming 100% horizontal dipole emitters (green emitting) in the EML. The yellow star with (ETL, HTL)=(65 nm, 195 nm) in (c) represents the structure with highest η_{filler} (~88.4%).

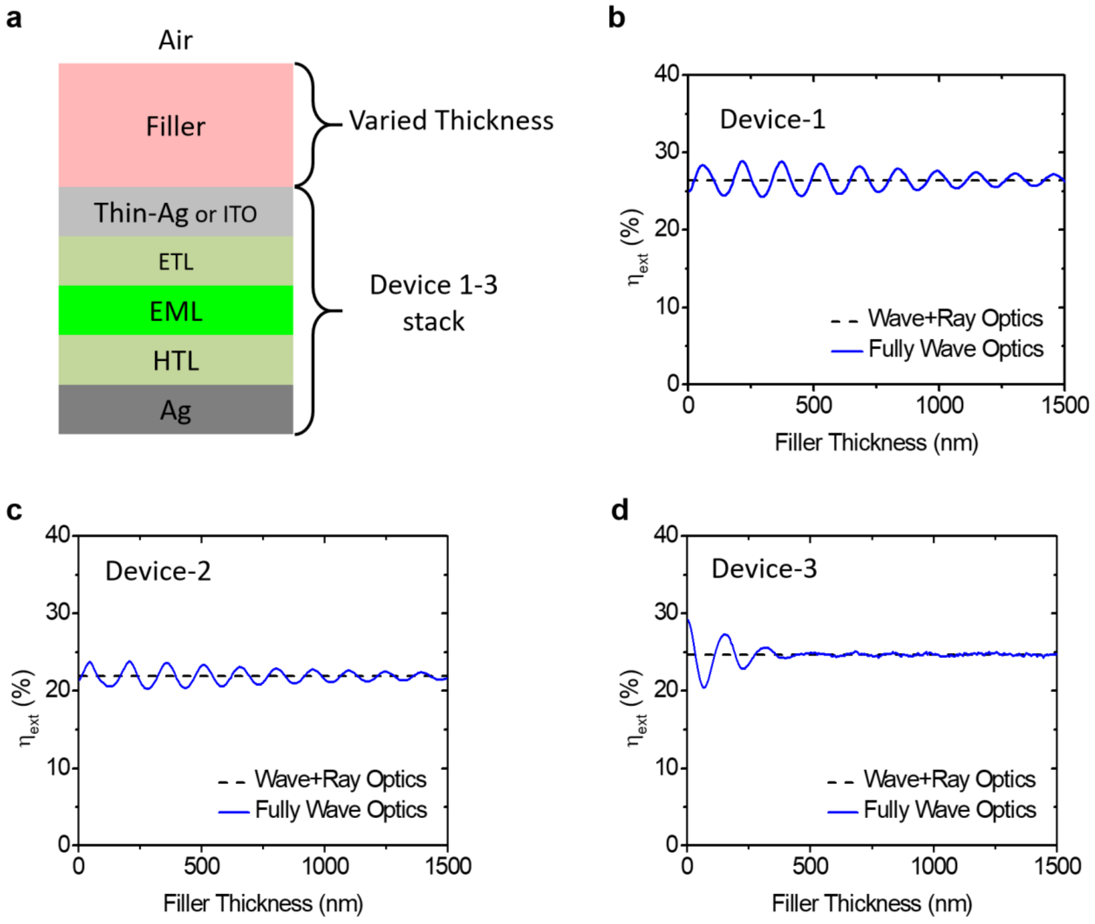

Figure S19. Validation of the multi-scale optical simulation (wave optics+ray optics). (a) The planar device configuration with the **device 1-3** OLED stack (green isotropic emitter) but different filler overcoating thicknesses used for the comparison/validation simulation study. **(b)-(d)** Comparison of η_{ext}'s calculated by (i) the fully rigorous electromagnetic wave optics approach that deals the OLED stack and the filler overcoating as a whole by wave optics, and (ii) the wave optics+ray optics approach that first uses the wave optics to calculate coupling of OLED internal emission into the filler layer (assumed semi-infinite) and then uses the ray optics to further calculate out-coupling from the filler to air: **(b) device 1** OLED stack, **(b) device 2** OLED stack, and **(c) device 3** OLED stack. η_{ext}'s calculated by both methods agree well within ±4.5%, ±3.5%, and ±1% deviation percentages for **devices 1**, **2**, and **3**, respectively (smallest for **device 3** having ITO as transparent top electrode for the filler overcoating thickness of >1 μm, indicating sufficient confidence in calculation of optical out-coupling efficiency in this work (which mainly deal with filler thicknesses of ≥1 μm).
